# Diffusion‐Based 3D Bioprinting Strategies

**DOI:** 10.1002/advs.202306470

**Published:** 2023-12-25

**Authors:** Betty Cai, David Kilian, Daniel Ramos Mejia, Ricardo J. Rios, Ashal Ali, Sarah C. Heilshorn

**Affiliations:** ^1^ Department of Materials Science and Engineering Stanford University 476 Lomita Mall Stanford CA 94305 USA

**Keywords:** bioprinting, diffusion, interfacial gelation, multi‐material constructs, perfusable structures

## Abstract

3D bioprinting has enabled the fabrication of tissue‐mimetic constructs with freeform designs that include living cells. In the development of new bioprinting techniques, the controlled use of diffusion has become an emerging strategy to tailor the properties and geometry of printed constructs. Specifically, the diffusion of molecules with specialized functions, including crosslinkers, catalysts, growth factors, or viscosity‐modulating agents, across the interface of printed constructs will directly affect material properties such as microstructure, stiffness, and biochemistry, all of which can impact cell phenotype. For example, diffusion‐induced gelation is employed to generate constructs with multiple materials, dynamic mechanical properties, and perfusable geometries. In general, these diffusion‐based bioprinting strategies can be categorized into those based on inward diffusion (i.e., into the printed ink from the surrounding air, solution, or support bath), outward diffusion (i.e., from the printed ink into the surroundings), or diffusion within the printed construct (i.e., from one zone to another). This review provides an overview of recent advances in diffusion‐based bioprinting strategies, discusses emerging methods to characterize and predict diffusion in bioprinting, and highlights promising next steps in applying diffusion‐based strategies to overcome current limitations in biofabrication.

## Graphical abstract

This review highlights an emerging class of 3D bioprinting strategies leveraging diffusion to control the structure and properties of printed constructs. These strategies involve the diffusion of specialized molecules into the printed ink, out of the printed ink, and/or within the printed construct. Using these strategies, complex tissue constructs, such as multi‐material or perfusable structures, can be fabricated.

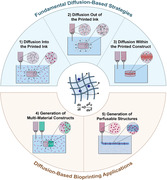

## 1 Introduction

The demand for highly specific in vitro models of human tissues, as well as tissue replacements for regenerative medicine, has necessitated new techniques to fabricate constructs recapitulating the structure and function of native tissues. As an extension of conventional additive manufacturing or 3D printing, 3D bioprinting has emerged as a promising method to pattern biomaterials and living cells into tissue‐like constructs. The main classes of bioprinting strategies include microextrusion, inkjet, laser‐assisted methods (e.g., two‐photon polymerization), and stereolithography,^[^ ^1^ ^]^ each with unique features and capabilities in fabricating constructs of various materials, geometries, and length scales. Furthermore, emerging technologies, such as volumetric bioprinting,^[^ ^2^, ^3^ ^]^ have been developed with the potential to rapidly fabricate large‐scale tissue constructs. In this review, we will focus on microextrusion bioprinting, as it is the most common and adaptable approach for the generation of scaffolds and cell‐laden hydrogel constructs.^[^ ^4^, ^5^ ^]^ In microextrusion bioprinting, a bioink, which consists of a mixture of polymeric materials and living cells, or a biomaterial ink, which consists only of acellular material components, is deposited layer by layer to generate a 3D construct.^[^ ^6^ ^]^ The compatibility of microextrusion bioprinting with a wide range of ink materials, coupled with its low cost and accessibility, makes it especially suitable for fabricating constructs with freeform designs and tunable mechanical and biological properties.^[^ ^5^, ^7^ ^]^

While diffusion within extrusion‐based, bioprinted constructs has been studied,^[^ ^8^, ^9^ ^]^ more recently, a variety of extrusion‐based bioprinting strategies have been developed that leverage diffusion as a fabrication parameter to modulate the properties and geometry of printed constructs. In general, diffusion is a naturally occurring physical process that describes the mass transfer of a molecular species toward a more homogeneous distribution. Historically, studies of such phenomena in biofabrication have been focused on the diffusion of oxygen to encapsulated cells.^[^ ^8^, ^9^, ^10^, ^11^ ^]^ Diffusion within the aqueous environment of a printed hydrogel construct is crucial for the supply of oxygen, nutrients, and growth factors to encapsulated cells, both for ensuring cell survival and for driving cells toward desired phenotypes. Besides designing for certain diffusional properties, however, bioprinting approaches are emerging which actively utilize diffusion mechanisms to modulate the properties and geometry of printed constructs. Such strategies represent a paradigm shift from considering diffusion as a design parameter for sufficient oxygen and nutrient supply to leveraging diffusion mechanisms as a key part of the fabrication process. These strategies may employ the diffusion of functional molecules, such as crosslinkers, catalysts, or viscosity‐modifying agents, either into or out of printed inks to modulate their chemical, structural, or mechanical properties. Besides altering construct properties, diffusion‐based methods have been developed to expand the capability of 3D bioprinting to generate biological structures with complex architectures, including multi‐material constructs as well as perfusable networks mimicking those in the vascular, lymphatic, or gastrointestinal systems.

**Figure 1:**
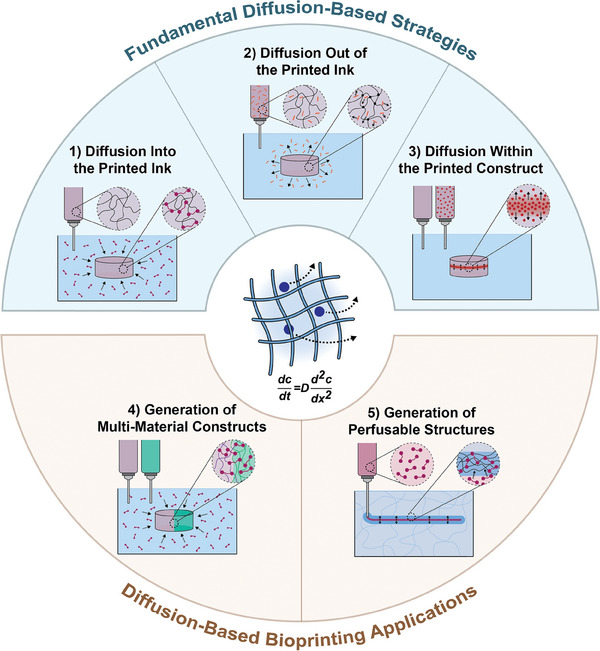
Summary of diffusion‐based bioprinting approaches and applications. Diffusion‐based bioprinting strategies can be subcategorized into those leveraging diffusion 1) into the printed ink, 2) out of the printed ink, and 3) within the printed construct. Applications of diffusion‐based bioprinting include 4) generation of multi‐material constructs via the interfacial diffusion of crosslinkers, and 5) generation of self‐supporting perfusable structures via diffusion‐induced gelation. For such approaches and applications, control of mass transport according to Fick's second law of diffusion (central schematic) is a key requirement.

Taking into account these recent developments, our review provides a novel perspective on this emerging category of bioprinting technologies, which we define as diffusion‐based bioprinting. This includes strategies to modulate ink printability and stability, to design functional bioinks and bioprinted constructs, as well as to create more complex and specialized structures using internal or interfacial diffusion patterns. First, we identify and summarize three fundamental strategies to modulate the properties of printed constructs based on the direction of diffusion: 1) strategies leveraging diffusion into the printed ink to modify construct properties and to stabilize the printed structure; 2) strategies leveraging diffusion out of the printed ink to engineer time‐dependent properties, and 3) strategies leveraging diffusion within the printed construct to establish localized gradients. Applying these fundamental strategies, we then discuss two types of applications leveraging diffusion‐based bioprinting to shape zonal interfaces and architectures in the fabrication of 4) multi‐material tissue constructs and 5) perfusable networks (**Figure** **1**). For each strategy and each application, we highlight recent advances, with a focus on novel ink formulations for extrusion‐based bioprinting that allow control over construct properties. As diffusion‐based bioprinting is a rapidly evolving strategy, we have not limited ourselves to examples that include living cells, but also highlight unique approaches which hold potential for live‐cell printing. In all of these approaches, understanding and controlling diffusion phenomena are key. Therefore, we next describe a variety of methods that have emerged to experimentally quantify diffusional properties as well as to predict and model diffusion processes in bioprinted constructs. Finally, we end with a discussion of how future advances in diffusion‐based bioprinting can help to overcome current limitations in biofabrication. Overall, we demonstrate why these advances in diffusion‐based bioprinting enable the fabrication of more complex and specialized tissue constructs and thus deserve strong recognition for future development.

## 2 Diffusion Into the Printed Ink: Controlling Printability and Construct Properties

The most common application of diffusion in bioprinting involves the inward diffusion of specialized molecules, such as crosslinkers, into printed inks. Such approaches have been developed to modify the properties of bioinks during or after the printing process, thus ensuring both bioink printability and construct stability. In extrusion‐based bioprinting, the bioink is required to possess certain rheological properties, such as paste‐like, shear‐thinning behavior enabling adequate extrusion.^[^ ^12^ ^]^ On the other hand, the final scaffold requires a different set of mechanical properties, such as a specific stiffness range or stress relaxation timescale, to regulate cell behavior in its intended application.^[^ ^13^, ^14^ ^]^ For instance, the stiffness of tissue constructs must be optimized to provide suitable biophysical cues guiding cell behavior, often similar to the stiffness of the native tissue to avoid mechanical mismatch for in vivo applications.^[^ ^15^ ^]^ To generate constructs with the desired properties while maintaining bioink printability, a variety of bioprinting strategies have been developed that leverage diffusion from solutions, support baths, or air into the printed ink to alter bioink properties.

### 2.1 Inward Diffusion for In Situ Gelation

In in situ gelation, the diffusion of molecules into the printed ink is employed to induce gelation during the printing process. Such methods may involve the extrusion of initially uncrosslinked or partially crosslinked inks into a crosslinker‐containing medium, allowing the diffusion of crosslinkers into the printed ink and thus an increase in the degree of crosslinking. Alternatively, a bioink and crosslinker solution may be extruded simultaneously using concentric nozzles.^[^ ^16^, ^17^, ^18^, ^19^, ^20^, ^21^ ^]^ Apart from methods involving crosslinker diffusion, we discuss other mechanisms for in situ gelation, such as self‐assembly or catalyst diffusion into the printed ink.

#### 2.1.1 Extrusion of Bioinks into Crosslinker‐Containing Medium

**Figure 2:**
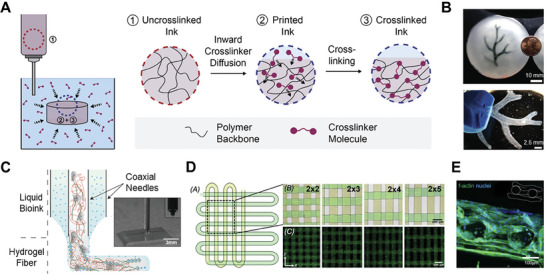
Bioprinting strategies involving the inward diffusion of crosslinkers for in situ gelation. A) Extrusion bioprinting into a crosslinker‐containing medium results in crosslinker diffusion into the printed ink, facilitating in situ gelation. B) A section of a human coronary arterial tree was fabricated by extruding alginate into a calcium ion‐containing support bath. Reproduced under terms of the CC‐BY license.^[^ ^25^ ^]^ Copyright 2015, The Authors, published by AAAS. C) Stable hydrogel fibers can be formed via coaxial bioprinting of a bioink in the inner nozzle and a crosslinker‐containing solution in the outer nozzle. Reproduced with permission.^[^ ^16^ ^]^ Copyright 2016, Wiley‐VCH. D) Scaffolds with different patterns were fabricated by coaxial printing of a GelMA‐alginate ink and calcium chloride solution. Reproduced with permission.^[^ ^18^ ^]^ Copyright 2017, Elsevier. E) A GelMA‐alginate scaffold containing endothelial cells (cytoskeleton in green; nuclei in blue) was fabricated by the coaxial extrusion of an endothelial cell‐laden GelMA‐alginate bioink and calcium chloride solution. Reproduced with permission.^[^ ^18^ ^]^ Copyright 2017, Elsevier.

In one diffusion‐based strategy, bioinks are printed into a crosslinker‐containing medium (i.e., air, solution, or support bath) for in situ crosslinking (**Figure** **2**A). During the printing process, crosslinkers present in the surrounding medium diffuse into the deposited filament, facilitating bioink gelation and conferring stability to the printed construct. In such cases, bioinks can either be printed in an uncrosslinked form or be partially crosslinked inside the print syringe prior to printing. For the printing of uncrosslinked bioinks, embedded bioprinting is commonly employed, in which inks are extruded into a viscoelastic support bath to improve ink printability and print fidelity.^[^ ^22^, ^23^, ^24^ ^]^ A notable example of this is the Freeform Reversible Embedding of Suspended Hydrogels (FRESH) method, where bioinks are extruded into support baths consisting of jammed gelatin microparticles. In FRESH printing, alginate bioinks were printed into support baths supplemented with calcium ions for diffusion‐based ionic crosslinking. Diffusion of ionic crosslinkers from the support bath into the bioink occurred during printing as well as subsequent incubation at 37 °C, through which the gelatin microparticle support bath was melted and removed (Figure 2B).^[^ ^25^, ^26^ ^]^

As compared to solution‐phase bioinks, bioinks that are in the gel phase both in the syringe and after printing may prevent cell sedimentation while reducing extrusion‐induced shear stresses by undergoing plug flow.^[^ ^27^, ^28^ ^]^ In such cases, bioinks may be extruded in a partially crosslinked form, then subjected to a secondary diffusion‐based crosslinking step immediately after the filament is deposited. As compared to first‐stage crosslinking, second‐stage crosslinking can occur by either identical or distinct crosslinking mechanisms. Dual‐stage crosslinking by a single mechanism may be performed with alginate, since it exhibits varying degrees of crosslinking based on the concentration of divalent cations. In one example, an alginate bioink containing a low concentration of calcium cations was extruded into a gelatin support bath with a higher calcium concentration.^[^ ^29^ ^]^ While the first‐stage‐crosslinked alginate may be readily extruded, secondary crosslinking based on calcium diffusion stabilized the construct, increasing the bioink stiffness by over one order of magnitude. This dual‐stage crosslinking system enabled neural progenitor cells (NPCs) within the bioink to be mechanically supported during printing while producing a stable construct suitable for NPC expansion and stemness maintenance.

Besides the dual‐stage ionic crosslinking of alginate, bioinks with two distinct crosslinking mechanisms, for which second‐stage crosslinking is based on diffusion from the surrounding medium, have been developed. In one demonstration, a Recombinant‐protein Alginate Platform for an Injectable, Dual‐crosslinked (RAPID) bioink was created by mixing alginate modified with proline‐rich peptide (P) domains together with an engineered protein, C7, which contained seven repeats of a complementary peptide (C) to enable first‐stage physical crosslinking. After extrusion into a calcium‐rich bath, second‐stage ionic crosslinking of alginate occurred based on the inward diffusion of calcium, leading to a stiffness increase of over two orders of magnitude.^[^ ^30^ ^]^

As opposed to crosslinker diffusion from a solution or support bath, in situ crosslinking can also be achieved via the direct extrusion of bioinks in air. The diffusion‐induced crosslinking of bioinks from crosslinker‐containing aerosols may enable greater oxygen transfer while allowing for lower crosslinker concentrations as compared to diffusion from solution, as shown for cell‐laden alginate bioinks crosslinked via diffusion from calcium chloride aerosols.^[^ ^31^ ^]^ A similar aerosol‐based crosslinking strategy was employed in a coaxial bioprinting system, where a cell‐laden collagen core was extruded simultaneously with an alginate sheath. The outer alginate layer was crosslinked in situ using a calcium chloride aerosol, serving to stably retain the collagen bioink within the printed structure.^[^ ^32^ ^]^ While aerosol‐assisted bioprinting enables effective crosslinking, it is less commonly employed compared to solution‐ and support bath‐based approaches due to the need for specialized, custom‐designed equipment.

Among the various printing media, bioink extrusion into a support bath possesses unique advantages, for instance enabling high shape fidelity with a variety of bioinks and providing greater control over the diffusion rates of encapsulated crosslinkers. In in situ crosslinking, the diffusion rate of crosslinkers into the bioink must be controlled to prevent overly rapid gelation, which may lead to issues such as clogging of the print nozzle or delamination between layers due to insufficient adhesion.^[^ ^33^ ^]^ While diffusion from solutions or aerosols is primarily influenced by the crosslinker concentration, the diffusional properties of crosslinkers within support baths can be controlled based on the support bath composition, porosity, and microstructure. In addition to bulk hydrogel materials, granular hydrogels, which consist of jammed microparticles, have been employed as support baths to allow greater control over their mechanical properties and microstructure. In such granular support baths, parameters such as microgel size, shape, and concentration can be varied to alter solute diffusivity.^[^ ^23^ ^]^ While embedded 3D printing enables greater control over crosslinker diffusion, careful consideration must be given toward possible interactions between crosslinkers and support materials, which may interfere with bioink crosslinking or alter the support bath rheological properties. For instance, multivalent cations such as calcium react with Carbopol, a support bath material composed of crosslinked acrylic acid polymers, greatly reducing its viscosity and storage modulus.^[^ ^34^ ^]^ As a result, the support bath for in situ crosslinking must be designed specifically for each bioink‐crosslinker combination to provide optimal rheological properties to support printed filaments as well as optimal diffusional properties to enable crosslinking.

#### 2.1.2 Coaxial Bioprinting of Bioinks with Crosslinker‐Containing Solution

Another common in situ crosslinking strategy is coaxial bioprinting, where a bioink and a medium containing crosslinkers are extruded simultaneously using a coaxial nozzle. This approach circumvents the challenges associated with crosslinker diffusion from a bulk medium by bringing the ink and crosslinker into direct contact during extrusion. To fabricate solid constructs, the bioink is extruded in the inner nozzle while the crosslinking solution is delivered in the outer nozzle, enabling in situ crosslinking of the bioink filament via inward diffusion of the crosslinker (Figure 2C). This strategy has been widely employed using alginate‐containing bioinks, which are extruded coaxially with a calcium chloride solution. During printing, alginate within the bioink is crosslinked via the inward diffusion of calcium ions. This in situ crosslinking mechanism increases bioink viscosity and prevents the collapse of printed filaments, producing constructs with precise patterns and high shape fidelity (Figure 2D). Furthermore, the diffusion of calcium ions across interfaces between filaments ensures cohesion between adjacent layers, thus enhancing the structural integrity of bioprinted constructs.^[^ ^16^ ^]^ In this strategy, alginate is typically combined with photo‐crosslinkable hydrogels, such as gelatin methacryloyl (GelMA), which are crosslinked by exposure to UV light, thus constituting a matrix for encapsulated cells (Figure 2E). This diffusion‐based coaxial bioprinting strategy has been employed with a variety of cell types, including endothelial cells and cardiomyocytes, myoblasts, and bone marrow‐derived mesenchymal stromal cells to fabricate cardiac‐, muscle‐, and cartilage‐like constructs, respectively.^[^ ^16^, ^17^, ^18^, ^19^, ^20^, ^21^ ^]^ In addition to solid structures, a similar coaxial bioprinting strategy, with the outer nozzle containing the bioink and the inner nozzle containing the crosslinking solution, has been applied to fabricate perfusable structures. Such methods are discussed in Section 6.1.

#### 2.1.3 Other In Situ Gelation Mechanisms

In addition to the diffusion of crosslinking reaction‐initiators, the in situ gelation of bioinks can be triggered by the diffusion of other molecules that facilitate crosslinking or self‐assembly. One common example is the gelation of collagen, which is soluble at low pH (i.e., below 5) but self‐assembles into fibers at neutral or basic pH (i.e., 6.5–8.5). Due to this pH sensitivity, the gelation of collagen bioinks can be induced via inward diffusion of a neutralizing buffer such as HEPES (4‐(2‐hydroxyethyl)‐1‐piperazineethanesulfonic acid). This was demonstrated in embedded bioprinting using the FRESH technique by extruding a collagen bioink into a HEPES‐containing gelatin microgel support bath (pH 7.4). Printed structures were incubated at 37 °C for at least 1 hour, enabling the inward diffusion of HEPES and resultant gelation of the collagen bioink.^[^ ^25^, ^26^ ^]^

**Figure 3:**
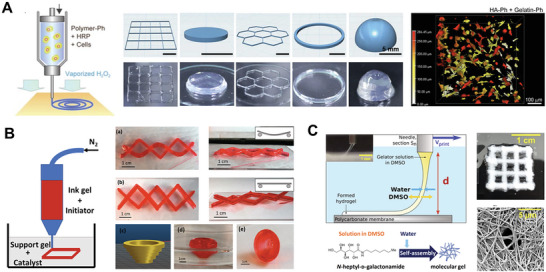
Bioprinting strategies involving the inward diffusion of molecules that trigger crosslinking or self‐assembly. A) A bioink containing polymers with phenolic hydroxyl moieties (polymer‐Ph) and horseradish peroxidase (HRP) was printed into air containing vaporized H_2_O_2_. During printing, H_2_O_2_ diffused into the ink and induced HRP‐catalyzed crosslinking, enabling the formation of stable constructs with a variety of geometries (left). Bioinks composed of hyaluronic acid (HA) and gelatin, both possessing phenolic hydroxyl (Ph) moieties, were printed using this peroxidase‐catalyzed technique. A 3D reconstruction of fluorescently labeled mouse fibroblasts encapsulated within the composite bioink showed cell spreading within the 3D matrix after three days of culture (right). Reproduced with permission.^[^ ^35^ ^]^ Copyright 2018, IOP Publishing. B) A Pluronic F127‐dimethacrylate ink was mixed with an initiator (APS) and extruded into a support bath containing a catalyst (TEMED). The diffusion of catalyst into the printed ink enabled in situ polymerization inside the support bath, allowing the fabrication of complex 3D constructs with high stability and shape retention. Reproduced with permission.^[^ ^36^ ^]^ Copyright 2017, American Chemical Society. C) A solution of amphiphiles in DMSO was extruded into a water bath. The diffusion of water into the printed ink facilitated rapid self‐assembly of amphiphiles into fibers, thus stabilizing the ink filament. Reproduced with permission.^[^ ^38^ ^]^ Copyright 2020, Elsevier.

Furthermore, in situ crosslinking can be induced via the inward diffusion of catalyst molecules. In one example, a bioink composed of polymers with phenolic hydroxyl (Ph) groups was extruded into air containing hydrogen peroxide (H_2_O_2_) (**Figure** **3**A). During printing, H_2_O_2_ diffused into the bioink and was consumed by peroxidase within the bioink to catalyze the crosslinking of Ph moieties. When combined with mouse fibroblasts, bioinks containing derivatives of hyaluronic acid (HA) and gelatin, both possessing Ph moieties, exhibited >90% cell viability as well as cell migration and spreading after three days of culture.^[^ ^35^ ^]^ In another case of catalytically induced crosslinking, a Pluronic F127‐dimethacrylate bioink loaded with an initiator, ammonium persulfate (APS), was extruded into a support bath containing a catalyst, tetramethylethylenediamine (TEMED). After printing, the catalyst was allowed to diffuse into the bioink, initiating free‐radical polymerization to form a robust 3D construct (Figure 3B).^[^ ^36^ ^]^ Overall, these catalytically induced gelation methods enable the bioprinting of a wide array of polymers, which can be combined to produce bioinks with multiple functions.

Another mechanism for in situ gelation involves the self‐assembly of amphiphilic molecules, which has been demonstrated for the case of peptide amphiphiles in ionic solution.^[^ ^37^ ^]^ In one example, self‐assembly of the printed ink was employed in the bioprinting of small amphiphiles whose gelation is triggered in contact with water. In this method, a solution of small amphiphiles in dimethylsulfoxide (DMSO) was extruded into a water bath, leading to rapid in situ gelation by diffusion of water into, and DMSO out of, the printed ink (Figure 3C).^[^ ^38^ ^]^ Despite the variety of gelation mechanisms used, all of these approaches enabled the design of highly extrudable inks as well as stable printed constructs with suitable mechanical properties for cell culture.

### 2.2 Inward Diffusion to Alter Crosslink Formation Post‐Printing

To enable a greater variety of crosslinking patterns than is possible with in situ crosslinking, strategies involving the crosslinking of constructs after printing have been developed. In single‐stage crosslinking, uncrosslinked or partially crosslinked structures may be crosslinked post‐printing by immersion in a crosslinker solution, such as a calcium chloride solution in the case of alginate.^[^ ^39^ ^]^ This approach requires bioink formulations with adequate mechanical properties to form a robust, self‐supporting structure for transfer into, or addition of, a crosslinker solution. Due to these material requirements, the post‐printing, diffusion‐induced gelation strategy is widely employed with composite bioinks, which are supplemented with viscosity enhancers such as hydroxyapatite and nanofibrillated cellulose.^[^ ^40^ ^]^ In such approaches, open porous lattice structures are typically printed to ensure the homogeneity of crosslinker diffusion, which is allowed to occur into each individual filament instead of into a larger bulk structure.

**Figure 4:**
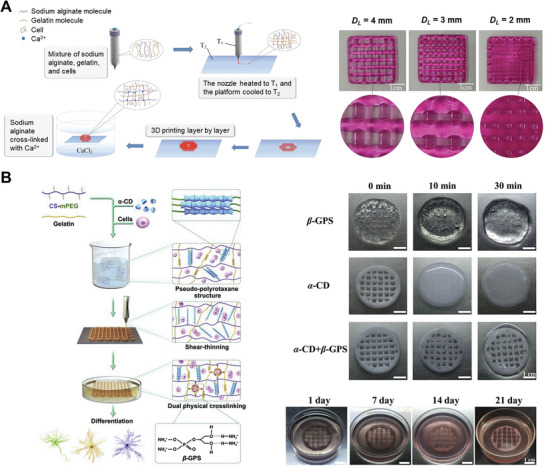
Bioprinting strategies involving post‐printing gelation through the inward diffusion of crosslinkers. Examples: A) A gelatin‐alginate bioink was extruded onto a cooled printing platform for the diffusion‐independent thermal gelation of gelatin. The construct was then transferred into a calcium chloride (CaCl_2_) solution, where the inward diffusion of Ca^2+^ led to second‐stage ionic crosslinking of alginate to form open‐porous scaffolds with varying infill patterns (strand‐to‐strand distances, *D_L_*: 2–4 mm). Reproduced under terms of the CC‐BY license.^[^ ^42^ ^]^ Copyright 2016, The Authors, published by Springer Nature. B) Bioinks composed of ɑ‐cyclodextrin (ɑ‐CD), pegylated chitosan (CS‐mPEG), and gelatin formed a supramolecular hydrogel inside the print syringe. After printing, constructs were transferred into a β‐glycerophosphate (β‐GPS) solution for second‐stage crosslinking. Dual‐stage‐crosslinked lattices retained their shape for 21 days, while lattices with only one stage of crosslinking (by either β‐GPS or ɑ‐CD) collapsed within 30 min. Reproduced with permission.^[^ ^46^ ^]^ Copyright 2020, KeAi Publishing.

Furthermore, multi‐component bioinks can be formulated to allow for first‐ and second‐stage crosslinking using separate mechanisms. For example, alginate has been combined with thermoresponsive materials, such as gelatin,^[^ ^41^, ^42^ ^]^ agar,^[^ ^43^ ^]^ or Pluronic.^[^ ^44^ ^]^ The resultant bioink exhibits first‐stage thermal gelation, enabling initial stabilization of the construct, and is then subjected to second‐stage ionic crosslinking to enhance its mechanical properties (**Figure** **4**A). As an alternative to thermal gelation, first‐stage covalent crosslinking can be employed in combination with second‐stage crosslinking through inward diffusion. In one demonstration, bioinks composed of amine‐containing polymers were covalently crosslinked within the print cartridge through the inclusion of a polyethylene glycol crosslinker (PEGX) that reacts with amines. After printing, second‐stage crosslinking was performed via treatment with solutions containing EDC (N‐(3‐Dimethylaminopropyl)‐*N*‐ethylcarbodiimide) and NHS (N‐Hydroxysuccinimide) for covalent crosslinking of gelatin‐containing bioinks, thrombin and calcium chloride for enzymatic crosslinking of fibrinogen‐containing bioinks, and calcium chloride for non‐covalent crosslinking of peptide amphiphile‐containing bioinks.^[^ ^45^ ^]^ Another interesting example of first‐stage non‐covalent crosslinking used a bioink composed of ɑ‐cyclodextrin (ɑ‐CD), polyethylene glycol (PEG)‐grafted chitosan, and gelatin, which exhibited guest‐host interactions between ɑ‐CD and PEG side chains. After printing, a second crosslinking step was performed in which the construct was treated with β‐glycerophosphate, which forms ionic and hydrogen bonds with chitosan and gelatin (Figure 4B). While constructs with only first‐ or second‐stage crosslinking were unable to maintain their shape after 30 min, constructs with both crosslinking steps remained stable in cell culture medium for at least 21 days.^[^ ^46^ ^]^

While the post‐printing gelation approach enhances the stability and mechanical properties of printed constructs, it may produce dimensional changes that must be considered in the print design. For example, the ionic crosslinking of alginate post‐printing may result in a loss of print definition due to swelling and deswelling behaviors.^[^ ^47^ ^]^ When immersed in calcium chloride solutions, printed alginate‐agar constructs were found to deswell, with the extent of shrinkage dependent on the calcium chloride concentration.^[^ ^43^ ^]^ In another study, alginate‐nanocellulose scaffolds crosslinked in calcium chloride solution exhibited swelling toward empty holes in the scaffold without significantly altering the overall print dimensions, indicating that dimensional stability upon crosslinking post‐printing is strongly influenced by the geometry of the printed construct.^[^ ^48^ ^]^

As opposed to facilitating crosslink formation, diffusion into printed constructs can also be used as a means of crosslink disruption. In the case of alginate bioinks, this is commonly performed via immersion of printed constructs in a solution containing calcium chelators such as ethylenediaminetetraacetic acid (EDTA). In one demonstration, alginate microfibers were formed through the coaxial extrusion of alginate with calcium chloride solution, then encapsulated into a photo‐crosslinkable GelMA scaffold. After photo‐crosslinking of GelMA, the hydrogel was immersed in EDTA solution, resulting in the dissolution of alginate microfibers and thus the formation of interconnected microchannels.^[^ ^49^ ^]^ In addition to alginate, diffusion‐induced crosslink disruption has been applied for gelatin‐chitosan constructs, which were immersed in sodium hydroxide to hydrolyze the gelatin or immersed in acetic acid to protonate and solubilize the chitosan.^[^ ^50^ ^]^ This diffusion‐based strategy opens up opportunities for the design of sacrificial inks to fabricate perfusable or hollow structures, although many of these crosslink disruption strategies have limited cell compatibility.

### 2.3 Inward Diffusion to Introduce Additional Functionality

**Figure 5:**
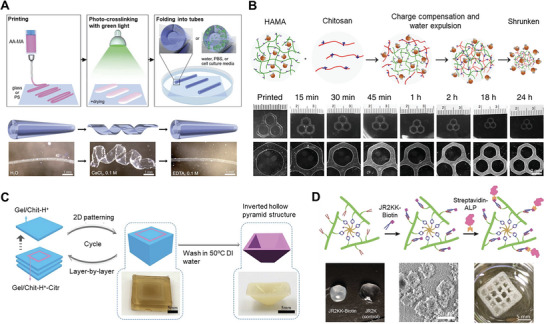
Bioprinting strategies leveraging inward diffusion to introduce additional functionality. A) Printed methacrylated alginate (AA‐MA) films were crosslinked by light irradiation, then transferred into an aqueous medium with or without ions for spontaneous folding into tubes. For printed AA‐MA films, reversible folding/unfolding behavior was achieved by immersion in calcium chloride solution to trigger unfolding, followed by immersion in EDTA to trigger re‐folding. Reproduced with permission.^[^ ^53^ ^]^ Copyright 2017, Wiley‐VCH. B) The diffusion of chitosan into printed methacrylated hyaluronic acid (HA‐MA) constructs resulted in shrinkage due to charge complexation. The degree of shrinkage was dependent on the immersion time in chitosan solution. Reproduced under terms of the CC‐BY license.^[^ ^54^ ^]^ Copyright 2020, The Authors, published by Elsevier Nature. C) The diffusion of citrate ions into printed sacrificial gelatin‐chitosan inks was used to form a non‐sacrificial double‐network hydrogel. An inverted hollow pyramid was fabricated by sequentially patterning gelatin‐chitosan and citrate inks. Reproduced with permission.^[^ ^50^ ^]^ Copyright 2020, IOP Publishing. D) Printed constructs composed of peptide‐conjugated HA were treated with complementary peptides, which diffused inward and subsequently associated with the hydrogel. To enable biomineralization, constructs were first incubated with a biotin‐labeled peptide (JR2KK‐Biotin), which recruited avidin‐modified proteins, then exposed to streptavidin‐modified alkaline phosphatase (streptavidin‐ALP) for ALP functionalization. The resultant ALP‐containing constructs exhibited mineral deposition in the presence of Ca^2+^. Reproduced under terms of the CC‐BY license.^[^ ^55^ ^]^ Copyright 2020, The Authors, published by IOP Publishing.

Apart from modulating the degree of crosslinking, inward diffusion has been employed to alter the shape, biofunctionality, and morphology of printed constructs. These strategies are considered as a subset of 4D bioprinting, where time is employed as the fourth dimension to enable stimuli‐responsive transformations. For instance, the immersion of printed constructs in aqueous solution has been used to transform 2D architectures into 3D morphologies based on their anisotropic swelling behavior.^[^ ^51^ ^]^ In such strategies, ion diffusion can be employed to modulate the shape‐morphing behavior of printed structures. In one demonstration, printed alginate‐methylcellulose structures were designed to deform upon immersion in calcium chloride solution due to a combination of differential swelling and crosslinking.^[^ ^52^ ^]^ Furthermore, ion diffusion has been used to alter the swelling behavior of printed methacrylated alginate (AA‐MA) or methacrylated hyaluronic acid (HA‐MA) films. In this strategy, printed films were photocrosslinked and then immersed in an aqueous medium, leading to self‐folding into tubes due to a crosslinking gradient along the film thickness. As compared to water, immersion of printed films in phosphate‐buffered saline (PBS) greatly reduced swelling due to charge screening by diffusing sodium ions, driving the formation of smaller tubes. For AA‐MA films, immersion in calcium chloride solution led to further reductions in swelling due to second‐stage ionic crosslinking. Furthermore, AA‐MA films can be designed to unfold when treated with calcium chloride solution and subsequently re‐fold when treated with the chelator EDTA, thus producing a reversible actuation behavior (**Figure** **5**A).^[^ ^53^ ^]^

Apart from anisotropic deformation, inward diffusion has been employed to induce isotropic shrinkage of printed constructs. In one demonstration, constructs composed of a hydrophilic polyionic polymer were immersed in a solution of oppositely charged polyions that diffuse into the construct to induce shrinkage through charge complexation (Figure 5B). Rapid, controllable shrinkage was demonstrated by immersing HA‐MA, HA‐MA–GelMA, or alginate constructs in chitosan solution, and the cytocompatibility of this process was demonstrated by the viability and proliferation of MCF‐7 breast cancer cells encapsulated in HA‐MA–GelMA constructs.^[^ ^54^ ^]^

In addition to altering construct geometry, inward diffusion has been used to alter the composition and microstructure of printed constructs. In one example, a polyacrylamide (PAAm) network was introduced into printed alginate‐agar hydrogels by immersion in a solution containing the monomer acrylamide, crosslinker NN′‐methylenebisacrylamide, and catalyst APS. After 24 h, the polymerization of acrylamide was allowed to occur via addition of the catalyst TEMED. Through the diffusion of monomer, crosslinker, and catalysts into the construct, a PAAm‐alginate double network was formed, enhancing hydrogel toughness by reinforcing the interfaces between printed stripes.^[^ ^43^ ^]^ Using a similar mechanism, a printed gelatin‐chitosan sacrificial material was converted into a non‐sacrificial double network hydrogel through the inward diffusion of citrate ions, which crosslinked chitosan electrostatically. Stable 3D constructs were formed by depositing an ink composed of sodium citrate and xanthan gum on top of printed gelatin‐chitosan layers (Figure 5C). After citrate crosslinking, the microstructure of the chitosan network was further modified via immersion in sodium hydroxide, which hydrolyzed gelatin and neutralized chitosan, reconfiguring the chitosan crosslinks to form crystalline network junctions.^[^ ^50^ ^]^

Besides crosslinkers, the diffusion of peptides has been employed to alter the mechanical properties and biofunctionality of printed constructs, thus affecting cell‐hydrogel interactions. In one example, hyaluronic acid (HA) was conjugated with a peptide designed to fold into a helix‐loop‐helix motif and dimerize into four‐helix bundles in the presence of Zn^2+^. These properties enabled the dynamic modulation of crosslinking density and hydrogel functionality via peptide folding. For instance, peptide‐conjugated hydrogels were printed into gelatin microparticle support baths with or without Zn^2+^, where peptide dimerization upon inward diffusion of Zn^2+^ drove the formation of more robust structures. Furthermore, the conjugated peptide is able to dimerize with complementary peptides with different functionalities, thus enabling the dynamic biofunctionalization of hydrogels. To control biomineralization, printed constructs were incubated with a biotin‐labeled complementary peptide, which was then associated with the construct through a diffusion‐limited process. The biotin‐labeled peptide in turn recruits and sequesters the enzyme alkaline phosphatase (ALP), facilitating biomineralization of the construct in the presence of Ca^2+^ (Figure 5D). This peptide‐mediated strategy was further generalized to a wider array of peptides, including those that include cyclic RGD peptide motifs to tune cell‐hydrogel interactions.^[^ ^55^ ^]^

Altogether, the above approaches highlight the versatility of inward diffusion to alter ink gelation and to introduce new functionalities. Examples of both of these categories of approaches are summarized in **Table** **1**. In bioprinting strategies based on inward diffusion, the compatibility of the biofabrication process with living cells is a key consideration. In some cases, the diffusing molecules may be harmful to cells encapsulated inside the printed ink, thus only allowing cells to be seeded onto the printed construct following removal of the diffusing species. A promising area of future development is to improve the cytocompatibility of diffusion‐based strategies through the design and incorporation of more cell‐friendly chemistries, enabling a wider variety of cell‐laden constructs to be fabricated.

**Table 1:** Bioprinting strategies based on inward diffusion to induce in situ gelation during printing. The inward‐diffusing agent(s) are underlined in each instance.

	Ink material	Printing medium	Gelation method(s)	Diffusion time	Cell type(s) included in ink	Ref.
**Extrusion**	Alginate (4%)	Gelatin particles + CaCl_2_ (0.1%)	Ionic	>1 h at 37 °C	N/a	[26]
	Fibrinogen (4%) + xanthan gum (0.2%)	Gelatin particles + thrombin (1 U mL^−1^)	Enzymatic	>1 h at 37 °C	N/a	
	Collagen (2.4%)	Gelatin particles + HEPES (50 mm)	pH	>1 h at 37 °C	HUVEC	
	Collagen (2.4%) + fibrinogen (2%)	Gelatin particles + thrombin (1 U mL^−1^)	Enzymatic	>1 h at 37 °C	Human embryonic stem cell‐derived CM	
	Alginate (4%) + HA (0.4%)	Gelatin particles + CaCl_2_ (11 mm)	Ionic	>1 h at 37 °C	N/a	[25]
	Collagen (0.89–0.96%)	Gelatin particles + HEPES (10 mm)	pH	>1 h at 37 °C	N/a	
	Fibrinogen (1%) + HEPES (10 mm)	Gelatin particles + thrombin (0.1 U mL^−1^)	Enzymatic	>1 h at 37 °C	N/a	
	Collagen (0.2%) + fibrinogen (1%) + Matrigel (0.025%) + HA (0.5%) + BSA (1%)	Gelatin particles + HEPES (10 mm) + thrombin (0.1 U mL^−1^)	pH + enzymatic	>1 h at 37 °C	MC3T3‐E1.4 fibroblasts, C2C12 myoblasts	
	Alginate (2%)	Gelatin + CaCl_2_ (8 mm)	Ionic	20 min at 37 °C	Murine hippocampal neural progenitor cells	[29]
	Tethered alginate (1%) + C7‐engineered protein (5%)	CaCl_2_ (100 mm) solution	Non‐covalent peptide bonds + ionic	10 min	NIH‐3T3 fibroblasts, hASC	[30]
	Alginate (0.5–4%)	CaCl_2_ (500 mm) aerosol	Ionic	30 s	NIH‐3T3 fibroblasts, MSC spheroids	[31]
	*N*‐heptyl‐D‐ galactonamide (2.5%) in DMSO	Water	Self‐assembly	Immediate	Human neural stem cells	[38]
	Pluronic F127‐dimethacrylate (0.033%) + TEMED (0.1%)	Pluronic F127 (0.025%) + APS (1%)	Free radical	Immediate	N/a	[36]
	HA‐phenolic hydroxyl (0.38%) + Gelatin‐phenolic hydroxyl (0.3%) + HA (0.75%) + horseradish peroxidase (5 U/mL)	H_2_ O_2_ (1 m or 16 ppm) aerosol	Oxidative	2 min	10T1/2 mouse fibroblasts	[35]
**Coaxial**	Core: collagen (2%) Shell: alginate (4%) + CaCl_2_ (0.5%)	CaCl_2_ (10%) aerosol	Ionic	Immediate	MG63 osteoblast‐like cells, hASC	[32]
	Core: alginate (4%) + GelMA (4.5%) + 2‐hydroxy‐4′‐(2‐hydroxyethoxy)‐2‐methylpropiophenone (0.2%) Shell: CaCl_2_ (300 mm)	Air	Ionic + UV	Immediate	HUVEC	[16]
	Core: alginate (4%) + PEG‐fibrinogen (1%) + Irgacure 2959 (0.01%) Shell: CaCl_2_ (300 mm)	Air	Ionic + UV	Immediate	HUVEC, iPSC‐derived CM	[17]
	Core: alginate (4%) + GelMA (4.5%) + Irgacure 2959 (25 mm) Shell: CaCl_2_ (300 mm)	Air	Ionic + UV	Immediate	HUVEC	[18]
	Core: alginate (2%) + GelMA (7%) + GelMA‐coated gold nanorods (0.01%) + 2‐hydroxy‐4′‐(2‐hydroxyethoxy)‐2‐methylpropiophenone (0.25%) Shell: CaCl_2_ (300 mm)	Gelatin (2%) + CaCl_2_ (11 mm)	Ionic + UV	Immediate	Cardiac fibroblasts, neonatal rat CM	[19]
	Core: alginate (4%) + PEG‐fibrinogen (0.8%) + Irgacure 2959 (0.1%) Shell: CaCl_2_ (300 mm)	Air	Ionic + UV	Immediate	C2C12 myoblasts	[20]
	Core: GelMA (6%) + alginate (4%); GelMA (6%) + alginate (4%) + chondroitin sulfate amino ethyl methacrylate (4%) +/‐ HA‐MA (0.5%) Shell: CaCl_2_ (300 mm)	Air	Ionic + UV	Immediate	Bone marrow‐derived MSCs	[21]

Abbreviations: HA, hyaluronic acid; HEPES, 4‐(2‐hydroxyethyl)‐1‐piperazineethanesulfonic acid; BSA, bovine serum albumin; DMSO, dimethyl sulfoxide; TEMED, tetramethylethylenediamine; APS, ammonium persulfate; GelMA, gelatin methacryloyl; PEG, polyethylene glycol; HA‐MA, methacrylated hyaluronic acid; HUVEC, human umbilical vein endothelial cell; CM, cardiomyocyte; MSC, mesenchymal stromal cell; hASC, human adipose‐derived stem cell; iPSC, induced pluripotent stem cell.

## 3 Diffusion Out of the Printed Ink: Engineering Time‐Dependent Properties Post‐Printing

Another way to tune the characteristics of bioprinted constructs is the diffusion of ink components out of printed strands, zonal compartments, or the entire construct. This outward diffusion strategy is commonly used to serve at least one of two functions: 1) tuning the mechanical properties of bioinks following extrusion, and 2) modulating biological functionality dynamically within the printed construct. Often these functions are achieved by designing diffusible ink components that either temporarily enhance or inhibit crosslinking. Alternatively, the diffusing component may modify the ink properties temporarily during the printing process without interfering with the crosslinking of other components. This category includes the release of temporary thickening agents that ensure stable strand deposition during bioink extrusion, maintain bioink homogeneity, and prevent premature gelation of the bioink in the print cartridge. Finally, the outward diffusion of bioactive molecules from the printed construct can be used to introduce biofunctionality to printed constructs in a time‐dependent and/or spatiallydefined manner.

### 3.1 Mechanical Properties

For extrusion‐based bioprinting without a support bath, high‐viscosity bioinks are needed to prevent filament deformation and collapse during printing. However, a high polymer content, which enables adequate viscosity, may result in final construct mechanical properties that are detrimental to cells during 3D culture. The constant need to balance the printability of the bioink and the cytocompatibility of the resulting environment has been summarized as the quest to identify an ideal “biofabrication window”.^[^ ^56^ ^]^

**Figure 6:**
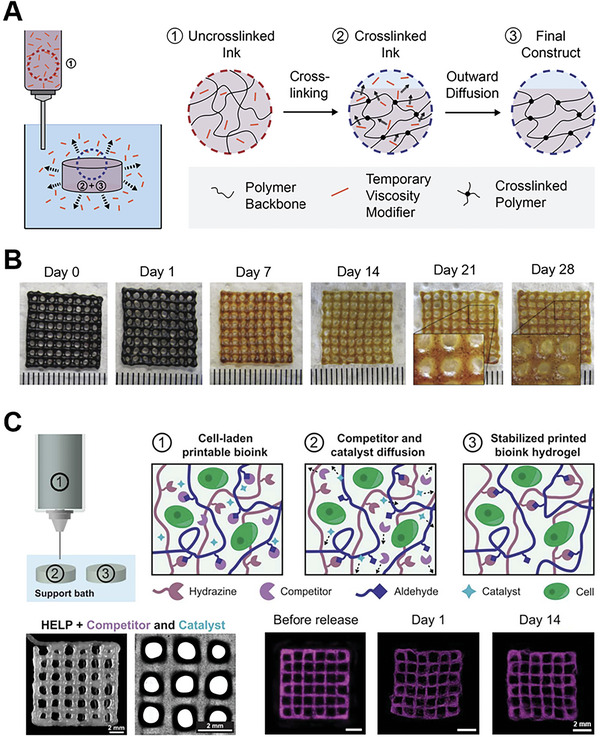
Methods leveraging outward diffusion to alter the properties of bioprinted constructs post‐printing. A) A temporary viscosity modifier can be included to improve ink printability and stability. After crosslinking of the printed ink, the viscosity modifier diffuses out of the construct. B) The diffusion of methylcellulose (MC) out of an alginate‐MC construct over time was observed by staining MC (dark violet) using a chlorine‐zinc‐iodine solution. Reproduced with permission.^[^ ^57^ ^]^ Copyright 2017, Wiley‐VCH. C) The mechanical properties of bioinks were modulated using small molecule catalysts and competitors, which diffused out of the bioink after printing to stabilize the printed construct. Hyaluronan and Elastin‐Like Protein (HELP) inks containing competitor and catalyst were printable while maintaining stability over two weeks. Reproduced under terms of the CC‐BY license.^[^ ^62^ ^]^ Copyright 2023, The Authors, published by AAAS.

To provide suitable mechanical properties for both printing and cell culture, the outward diffusion of temporarily viscosity‐modifying agents has been employed (**Figure** **6**A). In one example, suitable rheological properties for printing were achieved for an alginate bioink by blending in methylcellulose as a temporary viscosity‐enhancer. This was necessary as the included concentration of alginate (3%) is highly cytocompatible but, due to its low viscosity, does not allow the bioprinting of volumetric constructs without additional technical means such as a support bath or in situ crosslinking. In this strategy, methylcellulose stabilized the extruded bioink and diffused out to a large extent (>40%) after crosslinking the alginate using calcium chloride solution, leaving behind a micro‐porous, cytocompatible alginate network (Figure 6B). While it enables high printability, a key disadvantage of this approach is that the high viscosity of the bioink during extrusion leads to mechanical stresses on encapsulated cells. As a result, human mesenchymal stromal cells (hMSCs) encapsulated within the bioink exhibited a relatively low early viability (60–70%),^[^ ^57^ ^]^ while similar results were achieved for a comparable alginate‐methylcellulose blend with pre‐crosslinked alginate.^[^ ^58^ ^]^ To improve cell viability and spreading, the supplementation of alginate bioinks with human blood plasma,^[^ ^59^ ^]^ fibrillised collagen, or egg white‐derived albumin^[^ ^60^ ^]^ has been demonstrated. As opposed to methylcellulose as the viscosity‐enhancer, a similar approach employed Pluronic F127 in an alginate‐based bioink for the printing of hMSC‐laden bone and cartilage tissue substitutes. During and after crosslinking of alginate with calcium ions, Pluronic diffused out of the ink, leaving behind a microporous 3D alginate network with anisotropic microchannels generated by the inward diffusion of calcium.^[^ ^44^ ^]^

Although alginate is commonly used as a non‐sacrificial ink component, the outward diffusion of calcium ions may reduce ink stiffness and stability unless counteracted by the supplementation of calcium in the surrounding medium.^[^ ^61^ ^]^ Making use of the reversibility of ionic crosslinking, another study utilized alginate as a temporary thickening agent in a dual‐stage‐crosslinked alginate‐GelMA bioink. Alginate within the bioink was crosslinked in situ via coaxial extrusion of the bioink with a Ca^2+^ crosslinker solution, after which GelMA within the bioink was crosslinked using UV light. Alginate was then gradually released from the composite scaffold through the diffusion of calcium ions into the surrounding medium, resulting in pores that promote the spreading and proliferation of encapsulated endothelial cells.^[^ ^16^ ^]^ This combined inward‐outward diffusion strategy exemplifies how outward diffusion can be used to alter the microstructure and mechanical properties of constructs crosslinked based on inward diffusion.

As an alternative to bioinks crosslinked through reversible electrostatic bonds, bioinks with dynamic covalent bonds also possess viscoelastic properties similar to those of human tissue. Since these bioinks are already crosslinked inside the print syringe, the mechanical properties of the final construct are derived directly from the bioink. As a result, a key challenge for dynamic bioinks is to achieve suitable rheological properties for printing while producing constructs with sufficient stability for long‐term culture. In one example, both printability and stability were achieved for a dynamic HA and Elastin‐Like Protein (HELP) bioink through the use of small molecule modulators that diffuse out of the bioink after printing. In this system, a small molecule catalyst was included to increase the rate of dynamic bond exchange, while a small molecule competitor was used to reduce crosslinking by capping the chemical functional groups present in the bioink (Figure 6C). The inclusion of competitors and catalysts reduced bioink stiffness and increased shear‐thinning, respectively, enabling the extrusion of continuous filaments. After printing, the competitor and catalyst diffused out of the bioink, increasing the stiffness and stability of the construct.^[^ ^62^ ^]^

Overall, the kinetics of outward diffusion are strongly influenced by the mesh size and microstructure of the bioprinted construct. The mesh size and pore size of the hydrogel network are defined by the bioink morphology, which can be solid, fibrous, or granular (i.e., microgels), as well as the bioink composition, including the ratio of stable network component to diffusing component. The microstructure of the printed construct in turn determines its diffusional properties. Given these properties, quantitative experiments and computational models of outward diffusion (see Section 7) can be employed to predict the time‐dependent properties of the printed construct.

### 3.2 Bioactivity

Beyond the mechanical properties of printed constructs, biological activity is a property crucial to the functionality of bioprinted, cell‐laden tissue mimics. Diffusion can be leveraged to either induce or alter the bioactivity of printed constructs. As described in Section 2.3, printed constructs may be exposed to bioactive molecules that diffuse into the construct to induce certain biological functionalities. In contrast, the diffusion of enzymes out of printed constructs can be used to impart bioactivity to constructs after printing. In one example, the enzyme thrombin was entrapped in a photo‐crosslinked polyethylene glycol diacrylate (PEGDA) bioink. After printing, diffusion of thrombin out of the construct led to the formation of a cell‐laden fibrin biofilm upon contact with a cell‐laden fibrinogen solution.^[^ ^63^ ^]^ While the base structure was printed by digital light processing (DLP) in this example, this novel, diffusion‐based strategy could be readily applied to extrusion‐printed scaffolds. As an example of 4D printing, these systematic alterations to the scaffold structure over time introduce an important dimension to the bioprinting strategy.

While inward diffusion has been widely employed to modulate the properties of bioinks, substantially fewer examples of outward diffusion have been reported. As shown by the examples listed in **Table** **2**, outward diffusion can be employed with a variety of bioprinting methods and diffusing species to alter mechanical properties or introduce additional biofunctionality. Furthermore, these strategies are simple and typically do not introduce additional steps to the biofabrication process. Due to its ability to achieve time‐dependent properties, diffusion out of printed inks holds enormous potential in a variety of biological applications, including studies of the effects of microenvironmental changes on cell behavior.

**Table 2:** Bioprinting strategies based on outward diffusion to modify construct properties post‐printing.

Mechanism	Construct material	Printing method	Diffusing species	Property measured	Change in property	Cell type(s)	Ref.
MC diffusion out of the crosslinked alginate‐MC scaffold, leading to increased microporosity and reduced mechanical properties	Alginate (3%) + MC (9%)	Extrusion	MC	Elastic modulus in compression	101 ± 39 kPa (day 1); 77 ± 4 kPa (day 21)	hMSC (in bioink)	[57]
	Alginate (3%) + MC (0, 1, 3, or 9%)	Extrusion	MC	N/a	N/a	L929 fibroblasts (in bioink)	[58]
Diffusion of small molecule modulators out of printed dynamic covalent bioinks, increasing print stiffness and stability	HA‐aldehyde (0.5%) + HA‐benzaldehyde (0.5%) + ELP‐hydrazine (1%) + catalyst (10 mm) + competitor (10 mm)	Extrusion	Catalyst & competitor	Shear modulus	≈20% of 0 mm competitor control (immediately after printing); ≈100% of 0 mm competitor control (24 h after printing)	MCF10AT breast cancer cells, cancer‐associated fibroblasts (in bioinks)	[62]
Diffusion of Ca^2+^ from the printed construct into the surrounding medium, leading to reduced crosslinking and subsequent release of alginate	Alginate (4%) + GelMA (4.5%)	Coaxial	Ca^2+^ & alginate	Fluorescence intensity of fluorescently labeled alginate	≈20% of initial after 10 days	HUVEC (in bioink), neonatal rat CM (seeded post‐printing)	[16]
	Alginate (4%) + GelMA (4.5%)	Coaxial	Ca^2+^ & alginate	Pore size along construct borders	2.44 ± 1.24 µm initial; 7.33 ± 3.16 µm after alginate release	HUVEC =(in bioink)	[18]
	Alginate (2%) + GelMA (7%) + GelMA‐coated gold nanorods (0.01%)	Coaxial	Ca^2+^ & alginate	N/a	N/a	Cardiac fibroblasts, neonatal rat CM (in bioink)	[19]
	GelMA (6%) + alginate (4%); GelMA (6%) + alginate (4%) + chondroitin sulfate amino ethyl methacrylate (4%) +/‐ HA‐MA (0.5%)	Coaxial	Ca^2+^ & alginate	N/a	N/a	Bone marrow‐derived MSC (in bioink)	[21]
Fibrin biofilm formation upon contact with fibrinogen solution via crosslinking by diffusing thrombin	PEGDA + thrombin (50 U mL^−1^)	Light‐ based	Thrombin	Biofilm thickness	500 µm after 1 h incubation in 8% fibrinogen solution	NIH‐3T3 (in biofilm)	[63]

Abbreviations: MC, methylcellulose; HA, hyaluronic acid; ELP, elastin‐like protein; GelMA, gelatin methacryloyl; HA‐MA, methacrylated hyaluronic acid; hMSC, human mesenchymal stromal cell; HUVEC, human umbilical vein endothelial cell; CM, cardiomyocyte; MSC, mesenchymal stromal cell.

## 4 Diffusion within the Printed Construct: Establishing Defined Spatial Gradients

**Figure 7:**
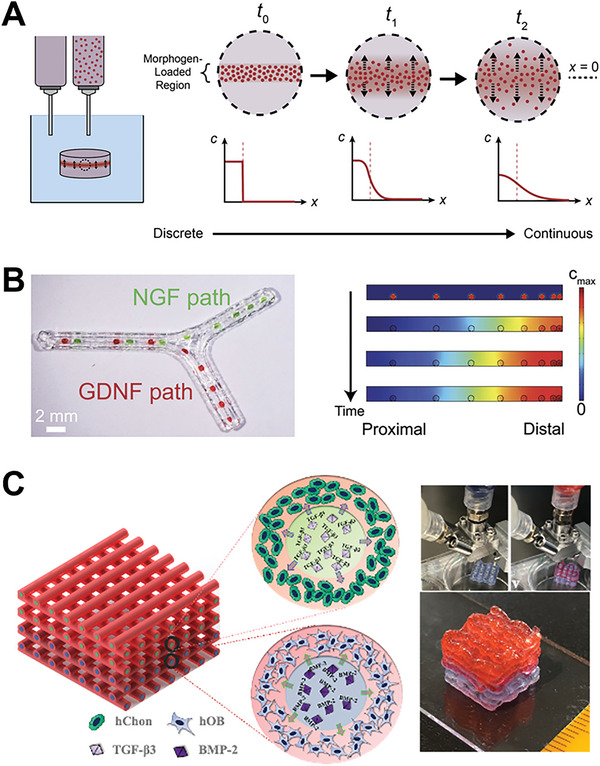
Bioprinting strategies leveraging diffusion within a printed construct. A) The diffusion of morphogens encapsulated within printed inks can be used to generate defined spatial gradients, which are initially discrete and become increasingly continuous over time within the printed construct. B) Spatial gradients of nerve growth factor (NGF) and glial cell line‐derived growth factor (GDNF) were generated by printing growth factor‐loaded hydrogels along a 3D‐printed nerve conduit (left). Diffusion of growth factors over time enabled the formation of continuous growth factor gradients, which were predicted by finite element modeling (right). Reproduced with permission.^[^ ^65^ ^]^ Copyright 2015, Wiley‐VCH. C) The differentiation factors transforming growth factor‐β3 (TGF‐β3) and bone morphogenetic protein‐2 (BMP‐2) were included in zonally defined core compartments of a core‐shell‐printed structure to induce chondrogenic and osteogenic differentiation of shell‐encapsulated human chondrocytes (hChon) and pre‐osteoblasts (hOB), respectively, by diffusion from the core depot to the shell. Using this locally restricted diffusion strategy, the co‐differentiation of different cell types in multi‐zonal constructs can be achieved. Reproduced under terms of the CC‐BY license.^[^ ^66^ ^]^ Copyright 2022, The Authors, published by IOP Publishing.

Apart from modulating construct properties through inward or outward diffusion, diffusion within a construct can be employed to generate defined spatial gradients (**Figure** **7**A). For example, morphogens, which are signaling molecules that induce a cellular response,^[^ ^64^ ^]^ are often incorporated into bioprinted constructs to direct cell behavior and phenotype with high positional specificity. While most bioprinting studies to date have focused on the direct patterning of morphogen gradients, leveraging diffusion to generate the desired spatial and temporal profiles has immense potential. Diffusion‐based strategies enable the generation of continuous morphogen gradients, which cannot be patterned directly through layer‐by‐layer bioprinting. By engineering specific diffusional patterns within bioprinted constructs, spatial gradients of morphogens can be established. In one instance, diffusion was employed to generate a continuous gradient of growth factors along the sensory and motor branches of a 3D‐printed nerve guidance conduit. To pattern two distinct morphogen gradients, GelMA hydrogels loaded with nerve growth factor (NGF) or glial cell line‐derived growth factor (GDNF) were printed at discrete points along the sensory and motor branches, respectively. A gradient of increasing concentration was generated by printing the morphogen‐loaded hydrogels more closely together toward the distal ends of each branch. After printing, diffusion within the construct enabled the formation of a continuous gradient of growth factors, which was predicted with finite element modeling (Figure 7B). The diffusive NGF gradient acted as a chemoattractant for sensory axons, while the GDNF gradient acted as a chemokinetic cue for enhanced Schwann cell migration.^[^ ^65^ ^]^

Diffusion within the construct can also be used to trigger biological functionality in bioprinted cells by ensuring a localized release of drugs or differentiation factors. In one strategy, the zone‐to‐zone diffusion of differentiation factors was used to support cell fate in a core‐shell‐printed osteochondral tissue model. Differentiation factors encapsulated within the core depot diffused into the cell‐laden shell, initiating the zonally defined differentiation of cell types encapsulated in the respective shell regions.^[^ ^66^ ^]^ This strategy ensures that active concentrations of differentiation factors can be kept within a zonally defined compartment of a multi‐layered and multi‐cellular scaffold, for instance inducing chondrogenic and osteogenic differentiation of chondrocytes and osteoblasts by the diffusion of transforming growth factor‐β3 (TGF‐β3) and bone morphogenetic protein‐2 (BMP‐2), respectively (Figure 7C). A Laponite nanoclay was used as a strong binding agent for differentiation factors to enable sustained release, resulting in a sustained effect over three weeks of culture. Different concentrations of Laponite led to adjustment of the release kinetics. While other approaches rely on inward diffusion of differentiation factors through capillary forces,^[^ ^67^ ^]^ this concept mainly relies on diffusion within the printed construct.

As an alternative to the direct patterning of morphogens, morphogen gradients can be generated within an initially homogeneous construct by designing specific diffusional patterns. In one demonstration, a gradient in β‐tricalcium phosphate (β‐TCP) was formed over time by bioprinting a layered scaffold with a gradient in porosity. In porosity‐graded constructs with a uniform initial β‐TCP concentration (10%), regions with higher porosity exhibited enhanced mineralization after four weeks. This enhanced osteogenic differentiation at later stages was hypothesized to occur due to the preferential diffusion of β‐TCP into higher‐porosity regions.^[^ ^68^ ^]^ By tailoring construct architecture to control diffusional patterns, such diffusion‐based approaches open up new opportunities for the generation of tissue constructs with heterogeneous cell populations.

Looking ahead, understanding and controlling diffusional patterns will be crucial to achieving temporal control of morphogen gradients. For in vivo tissue regeneration, the exposure of cells to morphogens at high doses may reduce therapeutic efficacy or even cause adverse effects, as documented for the osteoinductive growth factor BMP‐2.^[^ ^69^ ^]^ As a result, sustained spatial gradients in bioprinted constructs over days or weeks are often desirable. To engineer persistent gradients, the initial gradient magnitude, dimensions and architecture of the construct, and interactions between diffusing agents and printed inks must be optimized. For instance, the diffusion of growth factors can be slowed through the addition of binding agents, such as Laponite or nano‐hydroxyapatite, to the bioink.^[^ ^70^ ^]^ Alternatively, the onset of diffusion can be delayed by encapsulating molecules in delivery vehicles, such as polymeric microspheres.^[^ ^71^ ^]^ As a complicating factor, diffusion kinetics may be influenced by degradation or remodeling of the construct in vivo. Furthermore, the effects of bioink components on diffusion kinetics must be carefully evaluated, as common viscosity‐modifying agents, such as methylcellulose, have been shown to accelerate growth factor diffusion by increasing the extent of swelling.^[^ ^71^ ^]^ With an improved understanding of diffusion within complex environments, we anticipate that diffusion‐based methods will be applied to generate heterogeneous constructs with increasingly complex morphogen patterns to guide cell behavior in vitro and in vivo.

In addition to morphogen gradients to direct cell behavior, emerging approaches have employed diffusion within printed constructs to generate gradients of biomolecules to provide specialized functionalities. In one example, functional living scaffolds were fabricated by printing inks containing two types of core‐shell microgels, each encapsulating a different type of microbe. To generate specialized functionalities, such as ethanol fermentation, synergistic microbe types were employed, in which the conversion product of one microbe can be further processed by the other. Through the diffusion of this intermediate conversion product between the microgel cores, localized gradients were generated, leading to enhanced functional capabilities compared to direct mixing of the two microbe types.^[^ ^72^ ^]^ Looking beyond microbial interactions, the use of diffusion within printed constructs as a means to control interactions between multiple cell types holds promise for a wide range of biofabrication applications.

## 5 Generation of Multi‐Material Constructs

To fabricate more complex and functional constructs, a variety of 3D bioprinting strategies have been developed to pattern multiple materials and cell types into a single construct. Native tissues are dynamic composites of cells surrounded by a complex extracellular matrix (ECM), which provides physical scaffolding as well as signaling cues essential to cellular function. To mimic the heterogeneity of native tissues, each bioink should ideally be tailored for the cell type incorporated such that it presents the appropriate biochemical and biomechanical cues to promote cellular function. To achieve this goal, multi‐material bioprinting strategies enable the patterning of multiple cell types using customizable bioinks that guide cell‐material interactions post‐printing, promoting the dynamic biological evolution of printed constructs.

**Figure 8:**
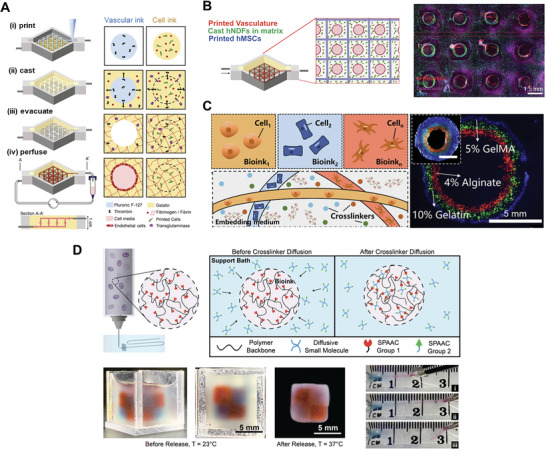
Bioprinting approaches leveraging diffusion to generate multi‐material constructs. A) Constructs with multiple cell types were fabricated by depositing a sacrificial (vascular) ink and a cell‐laden gelatin‐fibrinogen bioink, then casting a gelatin‐fibrinogen matrix over the printed inks. Crosslinking was facilitated by the diffusion of thrombin and transglutaminase from the cast matrix into the cell ink as well as the diffusion of thrombin from the vascular ink into the cast matrix. Reproduced with permission.^[^ ^73^ ^]^ Copyright 2016, National Academy of Sciences. B) A construct demonstrated the patterning of three different cell types, including human umbilical vein endothelial cells (HUVECs), human neonatal dermal fibroblasts (hNDFs), and human mesenchymal stromal cells (hMSCs) within the printed vasculature, cast matrix, and cell ink, respectively. Reproduced with permission.^[^ ^73^ ^]^ Copyright 2016, National Academy of Sciences. C) Multiple crosslinkers were included in a single embedding medium, allowing the fabrication of constructs integrating biopolymers with different crosslinking mechanisms. Reproduced with permission.^[^ ^74^ ^]^ Copyright 2022, IOP Publishing. D) UNIversal Orthogonal Network (UNION) bioinks enabled multi‐material bioprinting based on the diffusion of a small molecule crosslinker from a support bath into the printed structure. Cohesive structures composed of gelatin (red) and PEG (blue) were fabricated using the UNION strategy. Reproduced with permission.^[^ ^76^ ^]^ Copyright 2021, Wiley‐VCH.

For example, multi‐material constructs can be fabricated by the diffusion of enzymes between different regions of the print to facilitate crosslinking. In one strategy, a cell‐laden gelatin‐fibrinogen ink was printed along with a sacrificial vascular ink, and a cell‐laden gelatin‐fibrinogen matrix containing the enzymatic crosslinkers thrombin and transglutaminase was then deposited onto the printed inks (**Figure** **8**A). Following diffusion into the printed cell‐laden ink, thrombin facilitated rapid polymerization of fibrinogen into fibrin, while transglutaminase enabled gradual crosslinking of both fibrinogen and gelatin. Furthermore, the outward diffusion of thrombin in the sacrificial ink led to increased crosslinking in regions surrounding the ink filament, thus enhancing the stability of the vascular‐like network formed upon sacrificial ink removal. The embedded vascular network could then be injected with a suspension of endothelial cells, which lined the hollow channels. By including fibroblasts in the cast matrix, hMSCs in the printed cell‐laden ink, and endothelial cells within the vascular‐like network, constructs containing three different cell types were fabricated (Figure 8B).^[^ ^73^ ^]^

While the bioprinting of constructs with multiple cell types has been demonstrated, patterning multiple biopolymers into a single construct remains challenging due to the incompatible crosslinking mechanisms typically involved for different ink materials. Despite this challenge, the incorporation of different biopolymers in multi‐cellular constructs is necessary for tailoring bioinks to fulfill the matrix requirements of different cell types. To generate constructs from biopolymers with different crosslinking mechanisms, strategies employing the diffusion of multiple crosslinkers into printed inks have been proposed. In one example, a support bath composed of hydrophobically modified hydroxypropylmethyl cellulose and Pluronic F127 was employed for multi‐material bioprinting using multiple crosslinking mechanisms (Figure 8C). The support bath was designed such that the inclusion of various crosslinkers, including calcium chloride, sodium hydroxide, and transglutaminase, did not adversely affect its rheological properties. In contrast, the addition of PEG 400, a strong hydrophilic polymer, could be used to compromise the hydrophobic and hydrophilic associations between components, facilitating removal of the support bath from the printed construct. As an exemplary multi‐material construct, a tri‐layer hollow tube comprising an alginate inner layer, gelatin middle layer, and GelMA outer layer was fabricated via extrusion into a support bath containing calcium chloride and transglutaminase. Upon diffusion of crosslinkers into the printed inks, the alginate ink was crosslinked by calcium ions, while the gelatin and GelMA inks were crosslinked using transglutaminase. This approach was shown to be cell‐compatible, with endothelial cells encapsulated in printed GelMA structures exhibiting high viability (85–90%) and proliferation over ten days of culture.^[^ ^74^ ^]^

Apart from embedded bioprinting, coaxial bioprinting, as described in Section 2.1 above, can be used to generate dual‐material constructs by extruding a core containing a crosslinker that diffuses into the surrounding shell. In one example, a GelMA bioink containing calcium chloride was printed in the core and an alginate ink was printed in the shell. After crosslinking by calcium chloride diffusion, the alginate shell served to support and confine the core bioink, allowing for subsequent UV crosslinking. Various cell types, including breast cancer cells, fibroblasts, and endothelial cells, were encapsulated in the GelMA core with viabilities of 65–89% achievable.^[^ ^75^ ^]^

In integrating different biopolymers within a single construct, another key challenge is to ensure cohesion at the interface between different ink materials. This can be achieved by designing a universal crosslinking method compatible with multiple bioink materials, as opposed to using bioinks with different crosslinking mechanisms. Such crosslinking strategies would allow different bioinks to be crosslinked together at their interface, enhancing the structural integrity of the multi‐material construct. To achieve this goal, a UNIversal Orthogonal Network (UNION) crosslinking strategy was developed, where a small molecule crosslinker was encapsulated inside a support bath and diffused into bioinks conjugated with complementary bioorthogonal groups (Figure 8D). UNION bioinks were created with gelatin, HA, recombinant Elastin‐Like Protein (ELP), and PEG as backbone polymers. Due to their common crosslinking chemistry, multiple materials could be integrated into a unified, cohesive construct, as demonstrated for the combinations of PEG with gelatin and HA with ELP. Furthermore, the UNION crosslinking strategy is compatible with a variety of cell types due to its bio‐orthogonality. Cell types with different matrix requirements, including matrix‐adherent human corneal MSCs and non‐matrix‐adherent human induced pluripotent stem cell (hiPSC)‐derived neural progenitor cells, exhibited high viability and maintained their phenotype when cultured within UNION bioinks. Altogether, these results demonstrate the utility of the UNION strategy in fabricating multi‐material, multi‐cellular constructs with customizable bioink properties.^[^ ^76^ ^]^

As an alternative to the diffusion of crosslinkers, the diffusion of crosslinkable polymers into a cell‐laden matrix represents another strategy to generate multi‐material constructs dynamically during culture. In one demonstration, the bioprinting of dual‐material constructs was enabled by the diffusion of a photocrosslinkable gel precursor into a cell‐ or organoid‐containing Matrigel matrix. In this approach, termed hydrogel‐in‐hydrogel live bioprinting, two‐photon irradiation was used to initiate crosslinking of the gel precursor, enabling the creation of complex 3D patterns within the Matrigel construct. After this crosslinking step, the uncrosslinked gel precursor then diffuses out of the construct. This combined inward‐outward diffusion approach enables dual‐material constructs to be generated dynamically during culture, facilitating studies of the effect of matrix architecture and mechanical properties on cell behavior.^[^ ^77^ ^]^

Due to their ability to integrate multiple biopolymers into a single construct, diffusion‐based bioprinting strategies open up opportunities for the customization of bioinks for specific cell types. We anticipate that these strategies will be generalized toward new materials and crosslinking chemistries, thus expanding the library of compatible bioinks for creating more heterogeneous and biofunctional constructs.

## 6 Generation of Self‐Supporting Perfusable Structures

While the human body contains a variety of hollow structures, such as vascular networks, lymphatic vessels, and the gastrointestinal tract, the complexity of such structures has been challenging to replicate using biofabrication techniques. Over the past decade, diffusion‐based bioprinting methods have emerged that are uniquely capable of generating self‐supporting perfusable structures with high geometric complexity, precision, and tunability. These methods overcome key challenges associated with layer‐by‐layer extrusion, which typically results in hollow constructs that have an uneven surface prone to leakage due to gaps or delamination. To fabricate perfusable structures, two main classes of diffusion‐based bioprinting methods have been proposed: 1) coaxial extrusion of gel precursor shells and crosslinker‐containing cores; and 2) diffusion‐induced gelation at interfaces of printed inks with the surrounding medium. In this section, we provide case studies of such methods and their emerging applications.

### 6.1 Coaxial Printing of Perfusable Channels

**Figure 9:**
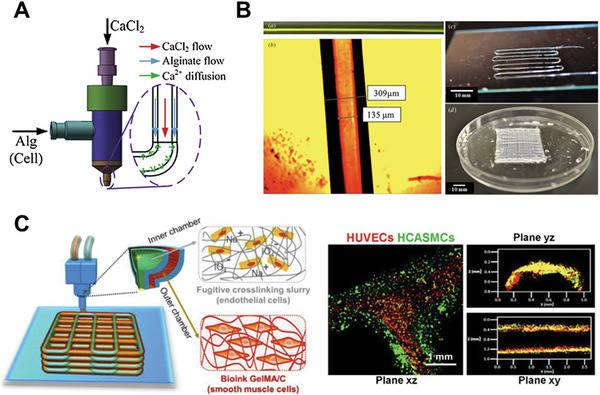
Diffusion‐based coaxial bioprinting strategies to fabricate self‐supporting perfusable structures. A) Perfusable channels were generated by co‐extruding a crosslinker solution in the inner nozzle and a hydrogel precursor in the outer nozzle. Reproduced with permission.^[^ ^78^ ^]^ Copyright 2014, Elsevier. B) Coaxial printing of a CaCl_2_‐containing core and an alginate shell was used to fabricate structures perfusable with yellow food dye. Both single‐ and multi‐layered perfusable structures were fabricated through continuous coaxial extrusion. Reproduced with permission.^[^ ^79^ ^]^ Copyright 2013, IOP Publishing. C) Channels incorporating two cell types were fabricated via the co‐extrusion of a core sacrificial ink containing crosslinker and endothelial cells (HUVECs, red) and shell bioink of catechol‐modified GelMA (GelMA/C) containing smooth muscle cells (HCASMCs, green). Reproduced with permission.^[^ ^81^ ^]^ Copyright 2019, IOP Publishing.

Coaxial bioprinting is an elegant method to induce in situ crosslinking of a bioink by simultaneous co‐extrusion with a crosslinking‐containing medium, as described in Section 2.2 for the fabrication of solid fibers. To achieve coaxial printing of perfusable channels, the core‐shell nozzle is used to extrude a filament with a hollow lumen, and the diffusion of crosslinker is used to stabilize the resulting channel. Diffusion may be designed to occur either within the print nozzle or within the filament immediately after extrusion. For example, coaxial bioprinting may be used to fabricate perfusable channels by extruding a gel precursor shell and a crosslinker‐containing core (**Figure** **9**A). This strategy is commonly applied by the co‐extrusion of an alginate shell with a calcium chloride‐containing core for in situ crosslinking.^[^ ^78^, ^79^ ^]^ Immediately after printing, calcium ions diffuse outward across the core‐shell interface, thus forming a crosslinked alginate shell (Figure 9B). The alginate ink can be doped with additives, such as carbon nanotubes^[^ ^80^ ^]^ or methacrylated HA,^[^ ^54^ ^]^ to modify the mechanical properties of the crosslinked channels.

In addition to utilizing the core compartment to deliver a crosslinker solution, the core can also be designed as a sacrificial bioink that includes cells along with the crosslinker. In one approach, a Pluronic F127 sacrificial ink containing an oxidative crosslinker was extruded in the core, and a catechol‐functionalized GelMA bioink was extruded in the shell. Diffusion of the crosslinker into the shell resulted in rapid oxidative crosslinking of the catechol‐modified bioink. During culture, the sacrificial ink in the core was gradually dissolved, thus depositing the cells contained within the core onto the channel surface. By including endothelial cells in the sacrificial core and smooth muscle cells in the gel precursor shell, vascular‐like constructs with encapsulated smooth muscle cells and a surrounding endothelium were fabricated (Figure 9C). As compared to coaxially printed alginate channels, the catechol‐modified GelMA channels exhibited greater cell adhesion and proliferation as well as enhanced structural stability after two weeks of perfusion.^[^ ^81^ ^]^ These results demonstrate the potential of diffusion‐based coaxial bioprinting strategies not only for the generation of perfusable channels, but also for the fabrication of patterned, multi‐cellular, tissue‐mimetic models.

### 6.2 Diffusion‐Induced Interfacial Gelation

While coaxial bioprinting can be used to fabricate channels with hollow lumens, the inner and outer diameters of printed structures are set by the nozzle geometry, precluding the fabrication of branched structures. To overcome this challenge, alternative strategies leverage diffusion mechanisms to generate perfusable structures without a core‐shell nozzle, with the print dimensions determined solely based on the printing parameters and diffusion time. In such strategies, diffusion at the liquid‐liquid, gel‐sol or gel‐gel interface of a printed structure and its surroundings is employed to enable gelation. In an early demonstration of this diffusion‐induced gelation concept, a sacrificial core of gelatin or Pluronic F127 loaded with Ca^2+^ was fabricated using a 3D‐printed mold, before multiple layers of alginate were formed by successive immersion into alginate solutions. GelMA inner layers were formed by including GelMA in the gel core; upon immersion of the gel core in an alginate solution containing a photoinitiator, a photo‐crosslinkable layer was formed via diffusion of the photoinitiator into the gel core. This method allows the freeform fabrication of perfusable multi‐layered vessels with up to seven layers, but requires manual post‐processing of the printed filament.^[^ ^82^ ^]^

Recently, diffusion‐induced interfacial gelation has been extended to bioprinting to generate perfusable networks with complex and customizable geometries. In one example, an interfacial diffusion printing (IDP) strategy was used for the one‐step fabrication of tubular vessels for application in cardiovascular tissue engineering. To enable in situ gelation, an ink composed of sodium alginate, bacterial cellulose, and acrylamide was extruded into a solution of calcium chloride and TEMED. Upon filament deposition, calcium chloride and TEMED diffused into the printed ink, initiating the ionic crosslinking of alginate and the free radical polymerization of acrylamide, respectively. After a set time for gelation, the uncrosslinked ink at the center of the filament was expelled to form a continuous, perfusable channel. The fabricated channels possessed suitable mechanical properties for implantation, demonstrating resistance against arterial pressure in a rabbit carotid artery model.^[^ ^83^ ^]^

**Figure 10:**
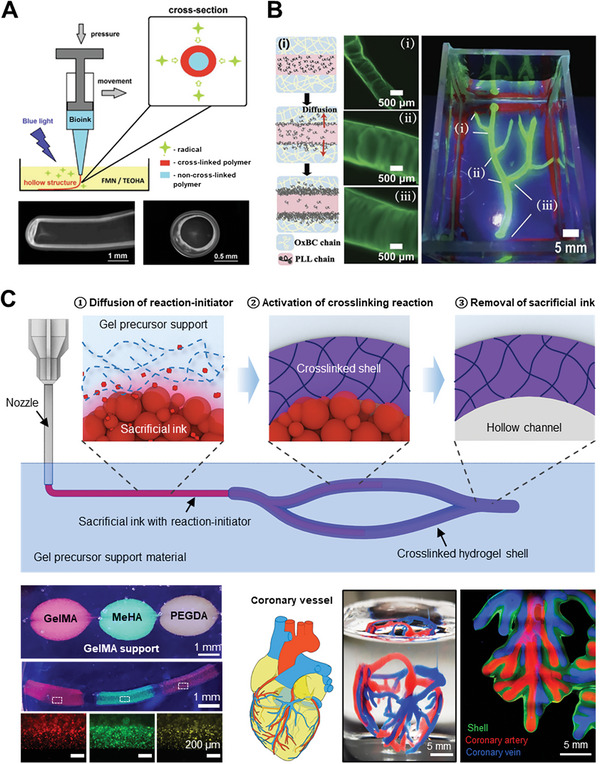
Diffusion‐based interfacial gelation methods to fabricate self‐supporting perfusable structures. A) Hollow structures were generated by extruding a photo‐crosslinkable bioink into a photoinitiator solution. Free radicals produced by a laser beam diffused into the printed filament, crosslinking an outer shell of the filament. Reproduced under terms of the CC‐BY license.^[^ ^84^ ^]^ Copyright 2021, The Authors, published by Frontiers Media S.A. B) A branched, perfusable network was fabricated by extruding a polylysine (PLL) ink into an oxidized bacterial cellulose (oxBC) solution. PLL diffused across the interface between the ink and oxBC solution, enabling the formation of complex coacervates. Reproduced with permission.^[^ ^86^ ^]^ Copyright 2023, Wiley‐VCH. C) In the GUIDE‐3DP strategy, a sacrificial ink containing a reaction‐initiator was extruded into a gel precursor support bath, after which the reaction‐initiator diffused into the gel precursor to enable crosslinking. GUIDE‐3DP is compatible with a variety of materials and enabled the fabrication of complex perfusable networks, such as a model of the coronary vasculature. Reproduced with permission.^[^ ^87^ ^]^ Copyright 2023, Wiley‐VCH.

In addition to small molecule crosslinking, the diffusion‐induced gelation method can also be applied to photo‐crosslinkable ink systems. In one study, channels with tunable diameters and wall thicknesses were fabricated by extruding a bioink composed of hyaluronic acid glycidyl methacrylate (HAGM) and PEGDA into a flavin mononucleotide/triethanolamine (FMN/TEOHA) photoinitiator solution (**Figure** **10**A). This technique, termed diffusion‐limited photofabrication, was based on the diffusion of free radicals generated by scanning a 450 nm laser beam across the photoinitiator solution. Upon bioink extrusion, the free radicals diffused from the solution into the filament, forming a crosslinked shell. The uncrosslinked bioink at the center of the filament, which has not been in contact with photoinitiators, can then be removed, leaving behind a hollow channel. While the outer diameter of the hollow fiber was controlled by the nozzle diameter, the inner diameter was adjusted via the amount of uncrosslinked bioink, which was defined by the light exposure dose and time. Human keratinocytes loaded in the bioink maintained high viability (>95%), thus demonstrating the cytocompatibility of the ink components and the fabrication process.^[^ ^84^ ^]^

Diffusion‐induced gelation can also be employed with self‐assembly processes to generate high‐resolution, perfusable channels. In one example, capillary‐like structures were produced based on the diffusion of graphene oxide (GO), which self‐assembles with an elastin‐like recombinamer, ELK1. A bioink consisting of ELK1 was extruded into a GO solution, resulting in the formation of a multi‐layered membrane upon spontaneous self‐assembly of the two components. The cytocompatibility of the material system was demonstrated using HUVECs seeded on sections of printed ELK1‐GO tubes.^[^ ^85^ ^]^

More recently, another aqueous‐in‐aqueous embedded bioprinting strategy leveraging the diffusion of an ink component was proposed for the generation of tailorable, branched network structures. In this approach, a polylysine (PLL)‐based bioink was printed into an aqueous solution of oxidized bacterial cellulose (oxBC) nanofibrils (Figure 10B). Through the outward diffusion of PLL, a complex coacervate membrane was formed at the interface between the ink and support bath due to the complexation of PLL, a weak polyanion, with oxBC, a polycation. HUVECs encapsulated within the bioink formed a confluent endothelial monolayer at the coacervated membrane, reducing the permeability of the vascular‐like network.^[^ ^86^ ^]^

While this complex coacervation approach demonstrates the potential of embedded bioprinting to fabricate complex perfusable networks, it is limited to material systems that have complementary charge. To overcome this challenge, a versatile strategy, termed Gelation of Uniform Diffusant in Embedded 3D Printing (GUIDE‐3DP), was developed for the fabrication of complex branched networks using a wide range of material systems (Figure 10C). In this approach, a sacrificial ink loaded with a crosslinking reaction‐initiator was extruded into a gel precursor support bath, which both stabilizes the printed filament and serves as the material eventually comprising the channel walls. After printing, the reaction‐initiator in the sacrificial ink diffuses uniformly into the surrounding gel precursor, forming a shell of crosslinkable material around the ink filament. Once crosslinking is complete, the sacrificial ink can then be removed, leaving behind a self‐supporting, hollow structure. This system was applied to a library of seven gel precursor support materials utilizing the diffusion of photoinitiators, enzymatic crosslinkers, or small molecules. Importantly, the GUIDE‐3DP strategy enabled precise control over the shell thickness by leveraging the predictable diffusion kinetics of reaction‐initiators. Separation of diffusion and crosslinking was achieved in the case of photo‐crosslinking, allowing the fabrication of more complex structures. Furthermore, GUIDE‐3DP allowed for the integration of cells in the fabricated structures, as demonstrated by the endothelialization of a branched, GelMA‐based network. Altogether, GUIDE‐3DP enabled the fabrication of complex perfusable networks based on commonly applied materials and equipment while enabling precise control over the channel dimensions.^[^ ^87^ ^]^

As summarized in **Table** **3**, perfusable structures can be generated by either coaxial extrusion or diffusion‐based interfacial gelation using a variety of bioinks and diffusants. In coaxial extrusion strategies, a core‐shell nozzle is necessary to form a hollow structure, whereas in diffusion‐induced interfacial gelation, hollow structures are generated solely by diffusion into or out of a printed ink filament. In both cases, the kinetics of diffusion are central to determining the dimensions of the tubular structures generated. Methods to examine and model diffusion are necessary to enable greater control over diffusion patterns and thus the dimensions of bioprinted perfusable structures. Examples of such methods are described in the following section.

**Table 3:** Bioprinting strategies leveraging diffusion‐induced gelation to fabricate perfusable structures.

	Ink material	Printing medium	Gelation method(s)	Diffusing species	Diffusion time [min]	Construct sizes	Cell type(s) in construct	Ref.
**Coaxial Printing**	Core: CaCl_2_ (2–5%) Shell: alginate (3–6%)	Air	Ionic	Ca^2+^ (outward)	Immediate	Wall thickness: 200–700 µm Outer diameter: 600–1300 µm	Cartilage progenitor cells (in shell)	[79]
	Core: CaCl_2_ (4%) Shell: alginate (3–4%) + multi‐walled carbon nanotubes (0.5–1%)	Air	Ionic	Ca^2+^ (outward)	Immediate	Avg. inner diameter: 461 ± 68 µm Avg. outer diameter: 930 ± 43 µm	Human coronary artery SMC (in shell)	[80]
	Core: CaCl_2_ (300 mm) Shell: alginate (0.5%) + HA‐MA (0.5–2.5%) or GelMA (5%) + Irgacure 2959 (0.5%)	Air	Ionic	Ca^2+^ (outward)	Immediate	Inner diameter: 100–700 µm Wall thickness: 50–400 µm Outer diameter: 200–1200 µm	N/a	[54]
	Core: Pluronic F127 (30%) + NaIO_4_ (23.4 nm) Shell: catechol‐modified GelMA (20%)	Air	Oxidative	NaIO_4_ (outward)	Immediate	Inner diameter: 500–1500 µm Wall thickness: 100–300 µm	Human coronary artery SMC (in shell) HUVEC (seeded post‐printing)	[81]
**Diffusion‐Induced Interfacial Gelation**	Alginate + bacterial cellulose + acrylamide	CaCl_2_ + TEMED	Ionic + free radical	Ca^2+^ & TEMED (inward)	1–4	Inner diameter: 0.5–3.5 mm Outer diameter: 2–5 mm	N/a	[83]
	Polylysine (0.5–3%)	Oxidized bacterial cellulose nanofibrils (0.5–1%)	Complexation	Polylysine (outward)	10–120	Inner diameter: 100–3000 µm Wall thickness: 2–16 mm	HUVEC (in bioink)	[86]
	Elastin‐like recombinamer ELK1 (2%)	Graphene oxide (0.1%)	Self‐ Assembly	ELK1 (outward); graphene oxide (inward)	0–1	Wall thickness: 2–150 µm Outer diameter: 0.5–2.5 mm	HUVEC (seeded post‐printing)	[85]
	HAGM (20%) + PEGDA (5%)	FMN (0.066 m) + TEOHA (33.5 mm)	Photo‐ Crosslinking	Free radicals (inward)	3–15	Wall thickness: 300–380 µm Outer diameter: 1–6 mm	Human keratinocytes (in bioink)	[84]
	Gelatin microgel (8%) + LAP (2 mm)	GelMA (10%) + Aristoflex AVC (2%)	Photo‐ Crosslinking	LAP (outward)	1–30	Wall thickness: 200–700 µm Outer diameter: 1–7 mm	HUVEC (seeded post‐printing)	[87]
	Gelatin microgel (8%) + LAP (2 mm)	PEGDA (5%) + Aristoflex AVC (2%)	Photo‐ Crosslinking	LAP (outward)	10	Wall thickness: 200–400 µm Outer diameter: 600–1600 µm	N/a	
	Gelatin microgel (8%) + LAP (2 mm)	HA‐MA (1%) + Aristoflex AVC (2%)	Photo‐ Crosslinking	LAP (outward)	10	Wall thickness: 200–300 µm Outer diameter: 600–1200 µm	N/a	
	Gelatin microgel (8%) + CaCl_2_ (1%)	Alginate (2%) + Aristoflex AVC (2%)	Ionic	Ca^2+^ (outward)	1–30	Wall thickness: 400–1200 µm Outer diameter: 2–3.5 mm	N/a	
	Gelatin microgel (8%) + thrombin (500 U mL^−1^)	Fibrinogen (5%) + Aristoflex AVC (2%)	Enzymatic	Thrombin (outward)	1–30	Wall thickness: 100–250 µm Outer diameter: 1–2 mm	N/a	
	Pluronic F127 (24%) + transglutaminase (1%)	Gelatin (10%) + Aristoflex AVC (2%)	Enzymatic	Transglutaminase (outward)	20	Wall thickness: 300 µm Outer diameter: 1.5 mm	N/a	
	Gelatin microgel (8%) + APS (20 mm)	Acrylamide (20%) + TEMED (20 mm) + bis‐acrylamide (0.1%) + Aristoflex AVC (2%)	Free radical	APS (outward)	5	Wall thickness: 150 µm Outer diameter: 1.2 mm	N/a	

Abbreviations: HA‐MA, methacrylated hyaluronic acid; GelMA, gelatin methacryloyl; TEMED, tetramethylethylenediamine; HAGM, hyaluronic acid glycidyl methacrylate; PEGDA, polyethylene glycol diacrylate; FMN, flavin mononucleotide; TEOHA, triethanolamine; LAP, lithium phenyl (2,4,6‐trimethylbenzoyl) phosphinate; AVC, Acryloyldimethyltaurate/VP Copolymer; APS, ammonium persulfate; SMC, smooth muscle cell; HUVEC, human umbilical vein endothelial cell.

## 7 Strategies to Characterize Diffusion in Bioprinting

With the growing complexity of diffusion‐based bioprinting methods, diffusion characterization and prediction have become increasingly crucial to controlling the properties and geometry of printed constructs. While the fundamentals of mass transport due to Fickian diffusion are well known and commonly taught within an engineering curriculum, how these fundamentals can be applied to bioprinting is a newly emerging topic. In this section, we will briefly provide an overview of some of the key fundamental concepts and then describe recent efforts to experimentally measure and computationally predict diffusion within bioprinted constructs.

### 7.1 Diffusion Fundamentals

Diffusional properties are commonly described by the characteristic diffusion coefficient *D* (also termed diffusivity), which corresponds to a specific diffusant‐environment combination. Historically, diffusion has typically been evaluated by creating a concentration gradient and measuring the resulting material flux. For a steady‐state system with a linear concentration gradient that does not change over time, the diffusion coefficient (often reported in units of µm^2^ s^−1^) can then be calculated using Fick's first law of diffusion:^[^ ^88^ ^]^

(1) $$J=\left.-D\left(\frac{\delta C}{\delta x}\right)\right|p,T$$

where *J* is the diffusion flux and *δC/δx* is the induced concentration gradient (at constant pressure, *p*, and temperature, *T*).

More frequently, the experimental system is not at steady state, resulting in a flux that changes over time. For these systems, one can track the concentration over time and/or at different locations and fit to Fick's second law of diffusion to determine the diffusion coefficient:^[^ ^88^ ^]^

(2) $$\frac{\delta C}{\delta t}=D\frac{\delta^{2}C}{\delta x^{2}}$$

according to which the change in concentration *C* over time *t* is proportional (by the diffusion coefficient *D*) to the change in concentration *C* at a given position *x*.

The diffusivity of a diffusing species through a bioink (or a support bath) will depend on the size of the diffusant and the properties of the surrounding medium. The diffusivity is commonly estimated using the Stokes‐Einstein relationship:^[^ ^89^ ^]^

(3) $$D=\frac{k_{B}T}{6\pi \mu R_{h}}$$

where *k*_B_ is Boltzmann's constant, *T* is temperature, *μ* is the viscosity of the surrounding medium, and *R*_h_ is the hydrodynamic radius of the diffusing molecule.

While the Stokes–Einstein relationship is based on diffusion through a liquid medium, in bioprinting, the medium is commonly a hydrogel. For these types of materials, the diffusivity is strongly influenced by the hydrogel morphology, which can take a variety of forms, including homogeneous, fibrous, or granular. For efficient diffusion through a homogeneous, continuous hydrogel, the diffusing species must be smaller in hydrodynamic radius than the mesh size of the polymeric hydrogel. This mesh size will depend on the polymer chain stiffness, polymer chain interactions with the solvent that may cause swelling, and the concentration of hydrogel crosslinks. The interested reader is directed to several excellent reviews and articles that discuss diffusion through homogeneous hydrogels.^[^ ^90^, ^91^, ^92^ ^]^ For granular hydrogels, the material is composed of jammed microparticles that are surrounded by a continuous network of voids. In these types of systems, the diffusing species will often move more efficiently through the solvent‐filled void space than through the gel‐based microparticles. As a result, the diffusivity within granular hydrogels is often controlled by altering the shape, size, and volume fraction of the microparticles, which in turn alters the geometry of the void space.^[^ ^93^ ^]^ Microgels are capable of enhancing diffusion substantially over bulk gels, as demonstrated for injectable GelMA microgels^[^ ^94^ ^]^ and norbornene‐functionalized hyaluronic acid microgels.^[^ ^95^ ^]^

In addition to hydrogel morphology, physicochemical interactions between the diffusant and the surrounding medium will also impact diffusivity. For example, designing electrostatic interactions, hydrogen bonding, or molecular recognition elements into a hydrogel can significantly slow down the rate of diffusion. These types of interactions have been widely used within the drug delivery community to achieve the controlled diffusion of many different bioactive molecules, and the interested reader is directed to several excellent reviews that describe these strategies.^[^ ^96^, ^97^, ^98^ ^]^ More recently, the use of dynamic covalent chemistry has also been used as a way to transiently tether diffusing species into a hydrogel.^[^ ^99^, ^100^ ^]^

Given the complexity of material and microenvironmental factors, characterization of diffusional parameters and properties is crucial to leveraging diffusion patterns for the fabrication of structures with defined architectures and specialized functionalities. In the next two sections, we describe both empirical and computational methods to characterize diffusion and highlight case studies of diffusion characterization and modeling in bioprinting.

### 7.2 Experimental Approaches

In the majority of diffusion‐based bioprinting studies, printed constructs are evaluated primarily based on their stability, measured in terms of their mechanical and physical properties,^[^ ^16^, ^74^, ^76^ ^]^ and their print fidelity relative to the intended structure.^[^ ^12^, ^101^ ^]^ For a thorough mechanistic understanding of diffusive effects, accurate measurements of the diffusional properties of printed constructs are crucial, although few bioprinting studies to date have incorporated this type of characterization.^[^ ^87^, ^102^ ^]^

**Figure 11:**
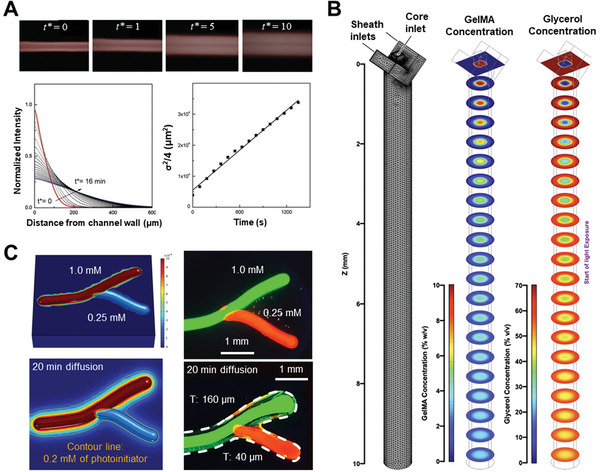
Experimental and computational characterization of diffusion in bioprinting. A) Fluorescence imaging was used to characterize the diffusion of a rhodamine‐based dye through a crosslinked Pluronic F127‐diacrylate hydrogel. The spatial peak variance *σ* ^2^ was calculated based on fluorescence intensity profiles, while the diffusion coefficient was determined from the slope of *σ* ^2^ as a function of time. Reproduced with permission.^[^ ^102^ ^]^ Copyright 2011, Wiley‐VCH. B) Finite element modeling was used to predict the diffusion of core and sheath inks inside a coaxial nozzle. The concentration profiles of GelMA, which diffused outward from the core to the sheath, and glycerol, which diffused inward from the sheath to the core, were determined at varying distances along the nozzle. Reproduced with permission.^[^ ^118^ ^]^ Copyright 2021, American Chemical Society. C) Finite element models were used to predict the diffusion of a photoinitiator from ink filaments printed into a GelMA support bath. The predicted concentration profiles were correlated with experimental results for a crosslinked bifurcated channel. Reproduced with permission.^[^ ^87^ ^]^ Copyright 2023, Wiley‐VCH.

The concentration of a diffusant is commonly determined using fluorescence‐based techniques. In one example, the diffusion coefficient of a rhodamine‐based dye in a crosslinked Pluronic F127‐diacrylate hydrogel was measured by injecting the dye into 3D‐printed microchannels. Fluorescence imaging was used to determine the fluorescence intensity around the channel lumen over time, normalized to a starting time point after dye injection. The spatial peak variance, *σ* ^2^, equal to the square of the diffusion length, was calculated from the diffusion profiles, and the diffusion constant was determined from a plot of *σ* ^2^ versus time (**Figure** **11**A).^[^ ^102^ ^]^

As an alternative to creating an experimental system, such as a microfluidic device, to create a macroscopic concentration gradient, modern techniques frequently use microscopic gradients to measure diffusive properties.^[^ ^103^ ^]^ A common microscopic method is fluorescence recovery after photobleaching (FRAP), which has been employed in bioprinting to evaluate diffusion within a variety of inks and support materials (**Table** **4**). In this method, a fluorescent molecule is incorporated into the sample, and a defined region is bleached by high‐intensity light irradiation. The rate of return of fluorescence signal, caused by the diffusion of unbleached molecules into the bleached region, is then measured to determine the diffusion coefficient according to Fick's second law. For example, FRAP was recently used to measure the diffusion coefficient of model FITC‐dextran molecules encapsulated in a printed ink within a GelMA support bath. The FRAP‐calculated diffusion coefficient was validated by measuring the diffusion profile in situ and comparing with predicted diffusion profiles.^[^ ^87^ ^]^

**Table 4:** Experimentally determined diffusion coefficients for representative inks and support materials in bioprinting.

Material	Diffusant	Diffusion coefficient [µm^2^ s^‐1^]	Characterization method	Ref.
Matrigel	FITC‐dextran (40, 500 kDa)	2.5 ± 0.4, 1.4 ± 0.1	FRAP	[77]
κ‐carrageenan microgel (CarGrow)	FITC (376 Da) Albumin‐FITC (66.4 kDa)	≈150 ≈30	FRAP	[115]
Cellulose nanofibers	FITC‐dextran (10, 20, 40, 70, 150, 250, 500 kDa)	130 ± 9, 105 ± 2, 82 ± 2, 85 ± 3, 82 ± 2, 63 ± 2, 39 ± 1	FRAP	[116]
Gelatin microgel (LifeSupport)	Diazide‐PEG‐FITC (600 Da) FITC‐dextran (20 kDa, 40 kDa)	87 ± 3 33 ± 2, 18 ± 2	FRAP	[76]
Pluronic F127 (26%)	Diazide‐PEG‐FITC (600 Da)	90 ± 40	Dialysis chamber	[76]
Gelatin‐BCN (6%)	FITC‐dextran (10 kDa)	≈50	FRAP	[76]
PEG‐BCN (6%)	FITC‐dextran (10 kDa)	≈65	FRAP	[76]
PEG‐BCN (8%)	FITC‐dextran (10 kDa)	≈65	FRAP	[76]

Abbreviations: FITC, fluorescein; PEG, polyethylene glycol; BCN, bicyclononyne; FRAP, fluorescence recovery after photobleaching.

While direct fluorescence imaging is the most common method to determine diffusivity in bioprinting studies, other in situ approaches to measure diffusivity within hydrogels have been developed. Another fluorescence‐based technique is fluorescence correlation spectroscopy (FCS), which measures the fluorescence intensity fluctuations induced by solute diffusion and quantifies the diffusion time based on an autocorrelation function. This method is able to measure the diffusivity of solutes at low concentrations, facilitating its use for studies of expensive solutes such as growth factors.^[^ ^104^ ^]^

Besides evaluating diffusion properties in the final construct, fluorescence‐based techniques have been applied to evaluate the diffusion behavior of crosslinkers in bioinks and support baths (Table 4). Within a certain medium, characteristic diffusion coefficients for the diffusing crosslinkers can be calculated to determine diffusion and crosslinking rates in the resulting bioprinted constructs. To study diffusion kinetics in the design of a new bioprinting strategy, termed UNION, diffusion coefficients of relevant molecules in gelatin microgel and Pluronic support baths were determined by FRAP or dialysis chamber methods, respectively.^[^ ^76^ ^]^ For a fluorescein (FITC)‐conjugated diazide‐PEG crosslinker (600 Da), similar diffusion coefficients were measured for gelatin microgel (87 µm^2^ s^−1^) and gel‐phase Pluronic (90 µm^2^ s^−1^) support baths. With increasing molecular weight, the diffusion coefficients of diffusants are expected to scale with the inverse of their hydrodynamic radius *R_h_*. Accordingly, diffusants with higher molecular weights (20 kDa and 40 kDa) exhibited significantly reduced diffusion coefficients (33 and 18 µm^2^ s^−1^) in a gelatin microgel medium due to their larger *R_h_* (3x and 5x, respectively). As a complicating factor, the mesh size and, therefore, diffusivity can change during gradual crosslinking of an extruded filament or a printed structure by diffusion from the surrounding medium.^[^ ^91^, ^105^ ^]^ This can make it more difficult to predict the resulting pattern or gradient. However, in the UNION bioinks, the mesh size was still sufficiently large after crosslinking to allow unhindered diffusion of molecules in the size of the crosslinkers (10 kDa dextran, *R*_h_ ≈ 2.3 nm; *D* ≈ 50–65 µm^2^ s^−1^ in different inks).^[^ ^76^ ^]^

In addition to measuring the diffusion coefficient, fluorescence imaging has been employed to assess the permeability of bioprinted channels with lumens seeded with endothelial cells. To assess the endothelial barrier function, the diffusion profile at various times after dye injection is determined using fluorescence imaging, and parameters such as the diffusional permeability^[^ ^73^ ^]^ or extent of diffusion into the surrounding hydrogel^[^ ^106^ ^]^ can be calculated based on changes in fluorescence intensity over time.

For all fluorescence‐based methods, a fluorescent diffusant is required. Since diffusion depends on the size of the soluble molecule,^[^ ^107^ ^]^ fluorescence approaches commonly employ a model molecule of a defined size (i.e., hydrodynamic radius) to mimic the dimensions and physical properties of the molecule of interest. Fluorescently tagged dextran polymers are often used as neutral model diffusants, since they are readily available in a range of sizes (i.e., molecular weights).^[^ ^108^ ^]^ Alternatively, if the size distribution of the molecule of interest is very wide, as is often the case for naturally derived biopolymers such as methylcellulose, the molecule of interest itself can be fluorescently tagged. However, control studies must be performed to ensure that the fluorescent probe does not significantly affect the diffusional properties. When the diffusant is expected to have strong interactions with the surrounding medium (e.g., through electrostatic interactions or hydrophobic interactions), a characterization method that does not rely on fluorescence may be preferred. For additional understanding of binding characteristics, zeta potential measurements and Langmuir binding models can be valuable tools.^[^ ^90^, ^109^ ^]^

Dynamic light scattering (DLS) is another powerful method to study the diffusion behavior of macromolecules in aqueous surroundings without the use of fluorescent molecules. For instance, it has been applied in hydroxyethycellulose gels to identify the diffusive patterns of sodium dodecyl sulfate (SDS) micelles.^[^ ^110^ ^]^ For larger‐volume samples requiring the non‐destructive bulk quantification of non‐fluorescent diffusants, newer methods have been developed that allow real‐time 3D monitoring of diffusion by optical coherence tomography (OCT),^[^ ^111^ ^]^ a combination of OCT and DLS,^[^ ^112^ ^]^ or indirectly by incorporating diffusant‐sensitive nanoparticles that can be tracked as components of the bioink.^[^ ^9^ ^]^

In some bioprinting methods, the outward diffusion of bioink components, drugs, or bioactive molecules is designed to occur into a surrounding medium over time. These methods allow direct assessment of the concentration of molecules released and thus their diffusional properties. Here, one can borrow the long‐standing techniques and analytical methods used by the drug delivery community. The direct assessment of drug release from hydrogels is especially beneficial since the diffusion kinetics often do not follow Fickian behavior.^[^ ^113^ ^]^ Unlike fluorescence imaging and DLS measurements, which are performed over short timeframes, these measurements can be used in long‐term release studies to determine transport kinetics over several weeks by spectroscopic methods, chromatographic methods, or enzyme‐linked immunosorbent assays.^[^ ^114^ ^]^

Depending on which diffusion‐based bioprinting strategy is being used, one may want to measure the diffusive properties of several different components within the system, and each measurement may require a slightly different technique. For example, diffusion through a biomaterial ink may change before and after crosslinking, since crosslinking typically results in a smaller mesh size that can hinder diffusion. Similarly, the speed that the nozzle travels through a support bath will alter the local viscosity, which may impact the local diffusion rate. In the case of zone‐to‐zone diffusion within multi‐material bioprinted constructs,^[^ ^66^ ^]^ the composition and morphology of each zone must be considered, since they may lead to significantly different diffusion rates. To improve our understanding of such complex bioprinted constructs, we expect that the use of existing techniques and the development of new methods to characterize diffusion within bioprinted systems will become more commonplace.

### 7.3 Computational Modeling

In addition to experimental methods to study diffusion characteristics, computational methods have been developed to predict and control diffusion in bioprinting. Besides the size and concentration gradient of the diffusing molecule, diffusion in bioprinted constructs is crucially dependent on the mesh size, morphology, and composition of the crosslinked hydrogel. The effects of these parameters can be predicted using multiscale models that account for different diffusion mechanisms depending on the scale of the solute (i.e., larger, similar, or smaller in size than the hydrogel mesh size).^[^ ^90^ ^]^

In bioprinting systems, diffusing molecules that are much smaller than the hydrogel mesh size are often of interest. In such cases, the resultant diffusion patterns are similar to those in other aqueous solutions and can be described by Fickian diffusion principles. Depending on the boundary conditions, construct geometries, and concentration gradients driving diffusion, Fickian‐based mass transport equations may be used to describe several diffusion scenarios encountered in bioprinting. When analytical solutions to Fick's laws of diffusion are not possible, finite element modeling (FEM) can be employed to understand and predict diffusion in bioprinting systems.^[^ ^117^ ^]^ In one example, the diffusion of core and sheath inks in coaxial bioprinting was assessed using a model coupling fluid flow and diffusion kinetics. In this system, a GelMA bioink was extruded in the core, while a glycerol solution was extruded in the sheath to confine the extruded GelMA filament. During extrusion inside the nozzle and before photo‐crosslinking, GelMA within the core diffused outward into the glycerol sheath, while glycerol diffused inward into the GelMA core. Accounting for these diffusion behaviors, FEM was used to determine the concentration profiles of GelMA and glycerol along the nozzle during extrusion (Figure 11B). The effects of different core and sheath concentrations and flow rates on the predicted concentration profiles were investigated to determine optimal printing parameters for stable filament formation.^[^ ^118^ ^]^

Besides diffusion inside coaxial nozzles, FEM has been employed in extrusion bioprinting to predict diffusion‐induced gelation. In one demonstration, the diffusion of graphene oxide (GO) into an elastin‐like recombinamer (ELK1) ink at different time points after extrusion was predicted using FEM. The predicted concentration profiles were used to validate the experimental results, which showed an increasing thickness of self‐assembled ELK1‐GO membranes with increasing diffusion time.^[^ ^85^ ^]^ In a more complex example, FEM was used to predict the diffusion of a photoinitiator contained within a gelatin microparticle ink into a photo‐crosslinkable GelMA support bath (Figure 11C). In this model, the concentration profiles of photoinitiator at different time points after extrusion were determined using diffusion coefficients obtained from experimental FRAP analysis. With increasing photoinitiator concentration or with increasing diffusion time, the thickness of the resultant crosslinked shell was predicted to increase. These FEM predictions were used to design a bioprinting process that could fabricate a bifurcated channel where the different channel branches had different shell thicknesses. The FEM‐predicted bioprinting strategies were then validated with experimental results. Altogether, these models serve to provide increased control over bioprinting processes and reduce the trial and error required for print optimization.^[^ ^87^ ^]^

In addition to predicting diffusion during the bioprinting process, computational modeling may be applied to predict time‐dependent changes in diffusion within bioprinted hydrogels, such as due to material degradation. As demonstrated for a non‐printed, protein‐loaded, degradable 4‐arm PEG acrylate‐dithiolglycolate hydrogel, Fickian diffusion during degradation can be modeled by considering how swelling and degradation affect the mesh size of the system. By fluorescence correlation spectroscopy, diffusion coefficients were determined in response to varying mesh sizes, enabling prediction of diffusibility for two model proteins, bovine serum albumin (BSA; 66 kDa) and immunoglobulin (IgG; 150 kDa).^[^ ^104^ ^]^ The complexity of such methods can be further enhanced by considering the contributions to degradation by encapsulated cells. For example, the diffusion of ECM components secreted by cells during tissue growth and subsequent enzymatic degradation was modeled in an artificial 3D tissue model based on an 8‐arm PEG‐norbornene hydrogel.^[^ ^119^ ^]^ In another study, time‐dependent diffusion properties were considered during four weeks of culture in tissue‐engineered cartilage consisting of alginate, agarose, gelatin, or fibrin gels for molecules with sizes of 3–500 kDa.^[^ ^120^ ^]^

In practice, experimental and computational methods are intricately linked: Experimental results provide input parameters for computational models, while computational methods can be used to predict and improve experimental outcomes. Novel software solutions, such as the open‐source package *PyFRAP*, are able to bridge the gap between experimental and computational approaches by fitting numerical simulations to experimental data.^[^ ^121^ ^]^ These efforts give rise to a new generation of characterization techniques as combinations of both experimental and computational methods.

## 8 Future Perspectives

Diffusion mechanisms have become an integral part of many bioprinting strategies, serving to increase the tunability and complexity of printed constructs. Such diffusion‐based strategies not only provide the basis for the crosslinking and stabilization of bioinks, but also can be leveraged to control structural features and to tailor the architecture of printed constructs. The diffusion phenomena involved in such processes occur on different time scales: Some strategies depend on diffusion‐based stabilization or softening within seconds or minutes^[^ ^45^, ^46^ ^]^ or use the diffusion time to define certain characteristics (e.g., channel thickness),^[^ ^87^ ^]^ while others rely on a sustained release over several weeks, as in the case of bioactive components (e.g., differentiation factors).^[^ ^66^ ^]^ With the varying time scales, functions, and applications of diffusing molecules, it may be possible to combine several distinct strategies into a single diffusion‐based biofabrication process. Looking forward, we expect that exciting developments in diffusion‐based bioprinting, including advancements in existing techniques and the development of novel strategies, will enable the fabrication of more complex and specialized tissue constructs. Quantifying and understanding diffusion mechanisms will be key to further leveraging diffusion kinetics in bioprinting strategies.

### 8.1 Leveraging and Controlling Diffusion Dynamics to Enhance Complexity in Biofabrication

Beyond advancements in existing bioprinting techniques, the complexity of printed constructs may be enhanced by integrating multiple diffusion strategies into a single fabrication process. Such hybrid diffusion strategies pave the way toward the fabrication of more complex tissue mimics. Through the careful design of diffusion strategies, complicated printing processes with multi‐channel extrusion systems, sophisticated technical equipment, or external stabilization techniques may be avoided. This was demonstrated in the case of diffusion‐induced interfacial gelation to fabricate perfusable networks, which removes the need for specialized equipment such as coaxial nozzles while maintaining control over channel patterns. Similarly, diffusion strategies may be used to generate mechanical or biochemical gradients in bioprinted constructs without the use of multiple extruders. The development of new cell‐compatible crosslinking strategies and material systems will undoubtedly contribute to the advancement of these approaches.^[^ ^76^ ^]^

As applied previously with the inward diffusion of crosslinkers coupled with the outward diffusion of a temporary viscosity enhancer,^[^ ^57^ ^]^ a combined inward‐outward diffusion approach has more recently been employed for tailoring cell‐ and organoid‐laden bioprinted constructs.^[^ ^77^ ^]^ Such strategies will enable the fabrication of constructs with enhanced biological functionality and geometric complexity. Furthermore, control over the mechanical properties of printed constructs can be improved by utilizing a wider library of crosslinkers, adjusting the crosslinker concentration and diffusion time, and applying secondary crosslinking steps. These strategies enable the mechanical properties of printed constructs to be modulated for mechanobiology studies of encapsulated or seeded cells in bioprinted constructs.

In addition to altering bioink properties, diffusion can be leveraged to introduce time‐dependent features to cell‐laden scaffolds for application as 3D in vitro models and implantable grafts. For example, diffusion enables the generation of defined gradients of growth factors and other biochemical or biomechanical cues within bioprinted constructs. This opens up opportunities for the creation of customized in vitro models to study cell behavior in response to signaling cues, as well as the incorporation of novel therapeutic features in implantable constructs. Furthermore, the outward diffusion of molecules within multi‐layered constructs can be designed to either 1) induce dynamic bioactive effects through the diffusion of bioactive molecules or 2) visualize material degradation through the diffusion of colored or fluorescently tagged diffusants. As a result, transferring previously described diffusion principles to multi‐layered constructs would be highly beneficial with potential applications in implantable constructs.^[^ ^122^ ^]^

In addition to extrusion‐based bioprinting, diffusion holds promise to become a key component of other bioprinting approaches, such as inkjet printing, volumetric printing, and DLP. These approaches can sidestep several of the limitations of microextrusion bioprinting, such as the confounding effects of infill patterns and outer geometries on diffusion kinetics. As a promising but less widely used approach, inkjet bioprinting enables cells, drugs, and growth factors to be patterned with high spatial precision.^[^ ^123^ ^]^ In inkjet printing, the diffusion of drugs or growth factors between droplets or zones can be employed to introduce biological functionality. In advanced light‐based approaches, such as volumetric bioprinting and DLP, the rapid diffusion and reaction of active species are requirements for the fabrication process. The viscosity of the photopolymer affects the diffusion of polymer chains to the interface, with a reduction in photopolymerization rate being observed above an optimal viscosity due to diffusion‐limited propagation.^[^ ^124^ ^]^ While the majority of these studies have not yet included cells, they hold great promise for future bioprinting advances. For example, volumetric bioprinting may be combined with microextrusion bioprinting for the fabrication of complex constructs such as perfusable channels^[^ ^3^ ^]^ and multi‐material structures.^[^ ^125^ ^]^ Gradients in light intensity can lead to gradients in crosslinking density within a construct,^[^ ^124^ ^]^ which could be used to design complex diffusion patterns in the future. To date, research on these emerging bioprinting modalities has mainly been focused on the characterization of diffusion during printing and within printed constructs, rather than utilizing diffusion as a fabrication parameter. However, diffusion‐based approaches hold great potential to be combined with these bioprinting modalities for the generation of constructs with controlled diffusion patterns or time‐dependent properties.

To better predict and tailor the structure and properties of bioprinted constructs, control over diffusion patterns is critical. Typically, diffusion occurs wherever a concentration gradient is generated, frequently at the interface of a bioink with a surrounding medium (i.e., air, solution, or support bath). The diffusion kinetics can be altered using various strategies, the simplest being altering the polymer content or mesh size. To provide greater control, diffusion‐selective hydrogels can be applied to induce directional diffusion.^[^ ^126^ ^]^ Another strategy to control diffusion is to employ diffusion barriers, such as a non‐diffusive material extruded from an additional cartridge, to prevent or reduce diffusion. Such barriers would enable concentration‐dependent properties, such as zonally defined mechanical,^[^ ^127^ ^]^ conductive,^[^ ^128^ ^]^ or paramagnetic^[^ ^129^ ^]^ characteristics, to be maintained over time.

Alternatively, diffusion may also be controlled by encapsulating molecules in vesicles such as liposomes or polymeric microgels. For instance, active molecules such as growth factors are commonly encapsulated in liposomes to delay the onset of diffusion and thus improve therapeutic efficacy in vivo.^[^ ^130^ ^]^ Similar approaches may be applied in bioprinting to design systems that delay diffusion, for instance enabling diffusants to be released in response to an external stimulus such as UV light. This could be particularly beneficial in diffusion‐induced gelation processes to avoid the immediate diffusion of reactive molecules during printing. Such strategies would enable the diffusion of active molecules to be initiated after the printing process is complete, such that a homogeneous diffusion pattern is achieved throughout the construct. If a heterogeneous yet defined diffusion pattern is preferred, the stepwise release of active molecules may be designed using multiple triggered systems.

In all of these developments, characterization and modeling of diffusion processes will facilitate the transfer of concepts to practice. By tuning the porosity, composition, viscosity, and intermolecular interactions, the diffusional properties of inks and support materials can be optimized. In addition to controlling the diffusional properties, a key next step is to obtain a better understanding of the diffusion kinetics across interfaces of inks with different media, such as solutions or support baths. Phase‐to‐phase diffusion can be enhanced using novel approaches such as chaotic bioprinting, which increases the interfacial area between compartments of different inks by simultaneous extrusion through a static mixer.^[^ ^131^ ^]^ To further enhance diffusion control, the adaptation of more advanced modeling approaches that have so far only been described for non‐printed tissue models to bioprinting will be critical.^[^ ^90^, ^121^, ^132^ ^]^

### 8.2 Biological and Clinical Applications of Diffusion‐Based 3D Bioprinting

By applying the diverse toolbox of diffusion‐based bioprinting strategies, highly specific in vitro tissue constructs may be generated to model disease mechanisms under defined conditions. To create more complex and functional in vitro models, a key aim is to design new methods of incorporating cells into bioprinted constructs. Currently, cells may be either encapsulated within the printed bioink or seeded onto the surface of constructs post‐printing. In the former case, which is desirable to achieve a homogeneous cell distribution, both the materials and crosslinking methods used must be compatible with living cells. Toward this end, bio‐orthogonal crosslinking strategies are being developed that are highly cell‐friendly and enable the recapitulation of cell‐cell interactions found in human tissue.^[^ ^76^ ^]^

One promising in vitro application of diffusion‐based bioprinting is the fabrication of perfusable structures to study the effect of vessel architecture (e.g., branching and curvature) on endothelial cell function. For instance, a diffusion‐induced interfacial gelation strategy has been employed to fabricate complex, endothelial cell‐laden networks mimicking vasculature.^[^ ^87^ ^]^ Such constructs can potentially be used as in vitro models of diseases such as vascular stenosis to study the impact of altered fluid dynamics on cell morphology and function. Furthermore, structures with patient‐specific geometries may be generated based on clinical imaging data,^[^ ^133^ ^]^ enabling the creation of bioprinted in vitro models or blood vessel substitutes. In addition to vascular mimics, the structures of all tubular organs of the lymphatic system, respiratory system, or gastrointestinal tract may be replicated using diffusion‐based interfacial gelation strategies.

Beyond in vitro models, some diffusion‐based bioprinting strategies have proven their suitability for in vivo environments. For instance, perfusable structures fabricated using diffusion‐based strategies have been investigated for use as small‐diameter vascular grafts. In one study, alginate‐based tubular structures fabricated using an interfacial diffusion strategy were implanted in a rabbit carotid artery by end‐to‐end anastomosis, leading to successful inflammation‐mediated integration.^[^ ^83^ ^]^ In such applications, a key challenge is to fabricate constructs with adequate mechanical properties for surgical handling and implantation (e.g., suturing). In addition to vascular grafts, the diffusion‐based generation of perfusable structures may be applied to fabricate other hollow implants, such as nerve guidance conduits.^[^ ^134^ ^]^ Furthermore, diffusion‐based bioprinting holds potential to enhance the therapeutic effect of such implantables by introducing features such as drug delivery, biodegradability, and patient‐specific and/or furcated geometries.

As bioprinted constructs become increasingly complex, interfacial tissue interactions are expected to become more commonplace. As a result, cell‐to‐cell and cell‐to‐matrix signaling are expected to become increasingly important, engendering increased demand for complex diffusion models involving various transported and interacting bioactive factors. On the other hand, heterogeneous, anisotropic structures are required to mimic interfaces between different tissue types with individual histologies, mechanical properties, and cell types. Such challenges can be readily solved by applying diffusion‐based strategies to generate growth factor gradients and intersectional tissue characteristics. As such, diffusion‐based bioprinting methods hold immense potential for a variety of tissue engineering applications.

## 9 Conclusion

While diffusion mechanisms play an important role in extrusion‐based bioprinting, the significance of diffusion phenomena has often not been fully acknowledged. Our review identifies three categories of diffusion‐based fabrication strategies based on directionality of diffusion and highlights two emerging applications for leveraging diffusion to fabricate more complex and specialized tissue constructs. In bioprinting approaches, the diffusion of specific molecules can be designed to occur either into or out of printed inks to modulate the structure, mechanical properties, or biological functionality of printed constructs. Moreover, diffusion can occur within the printed construct to shape its internal morphology and structural characteristics. These diffusion strategies have greatly expanded the applications of bioprinting, including the fabrication of more complex shapes, multi‐material constructs, and perfusable structures mimicking human tissues. Looking forward, advancements in bioprinting technologies, such as coaxial or multi‐material printing systems, open up new opportunities for leveraging diffusion to enhance the functionality of printed constructs. The development of new methods to control diffusion patterns, such as diffusion‐selective barriers or triggered release systems, will be crucial to i the capability of diffusion‐based bioprinting strategies. Furthermore, improvements in the characterization and modeling of diffusion patterns will allow the properties and geometry of bioprinted constructs to be more precisely controlled. Altogether, the use of diffusion in bioprinting holds great promise for the fabrication of complex tissue constructs with tailored architectures and dynamic functionalities.

## Conflict of Interest

The authors declare no conflict of interest.

## References

[1] S. Derakhshanfar , R. Mbeleck , K. Xu , X. Zhang , W. Zhong , M. Xing , Bioact Mater 2018, 3, 144. 29744452 10.1016/j.bioactmat.2017.11.008 PMC5935777

[2] P. N. Bernal , P. Delrot , D. Loterie , Y. Li , J. Malda , C. Moser , R. Levato , Adv. Mater. 2019, 31, 1904209. 10.1002/adma.201904209 31423698

[3] G. Größbacher , M. Bartolf‐Kopp , C. Gergely , P. N. Bernal , S. Florczak , M. de Ruijter , N. G. Rodriguez , J. Groll , J. Malda , T. Jungst , R. Levato , Adv. Mater. 2023, 35, 2300756. 10.1002/adma.202300756 37099802

[4] S. V. Murphy , A. Atala , Nat. Biotechnol. 2014, 32, 773. 25093879 10.1038/nbt.2958

[5] Y. S. Zhang , G. Haghiashtiani , T. Hübscher , D. J. Kelly , J. M. Lee , M. Lutolf , M. C. McAlpine , W. Y. Yeong , M. Zenobi‐Wong , J. Malda , Nat. Rev. Methods Primers 2021, 1, 75.

[6] J. Groll , J. A. Burdick , D.‐W. Cho , B. Derby , M. Gelinsky , S. C. Heilshorn , T. Jüngst , J. Malda , V. A. Mironov , K. Nakayama , A. Ovsianikov , W. Sun , S. Takeuchi , J. J. Yoo , T. B. F. Woodfield , Biofabrication 2018, 11, 013001. 30468151 10.1088/1758-5090/aaec52

[7] D. Chimene , R. Kaunas , A. K. Gaharwar , Adv. Mater. 2020, 32, 1902026. 10.1002/adma.201902026 31599073

[8] L. Figueiredo , C. Le Visage , P. Weiss , J. Yang , Polymers 2020, 12, 1260. 32486307 10.3390/polym12061260 PMC7361700

[9] E. Trampe , K. Koren , A. R. Akkineni , C. Senwitz , F. Krujatz , A. Lode , M. Gelinsky , M. Kühl , Adv. Funct. Mater. 2018, 28, 1804411.

[10] A. Erdem , M. A. Darabi , R. Nasiri , S. Sangabathuni , Y. N. Ertas , H. Alem , V. Hosseini , A. Shamloo , A. S. Nasr , S. Ahadian , M. R. Dokmeci , A. Khademhosseini , N. Ashammakhi , Adv. Healthcare Mater. 2020, 9, e1901794. 10.1002/adhm.201901794 PMC7500045 32548961

[11] R. K. Jain , P. Au , J. Tam , D. G. Duda , D. Fukumura , Nat. Biotechnol. 2005, 23, 821. 16003365 10.1038/nbt0705-821

[12] A. Schwab , R. Levato , M. D'Este , S. Piluso , D. Eglin , J. Malda , Chem. Rev. 2020, 120, 11028. 32856892 10.1021/acs.chemrev.0c00084 PMC7564085

[13] A. J. Engler , S. Sen , H. L. Sweeney , D. E. Discher , Cell 2006, 126, 677. 16923388 10.1016/j.cell.2006.06.044

[14] O. Chaudhuri , L. Gu , D. Klumpers , M. Darnell , S. A. Bencherif , J. C. Weaver , N. Huebsch , H. Lee , E. Lippens , G. N. Duda , D. J. Mooney , Nat. Mater. 2016, 15, 326. 26618884 10.1038/nmat4489 PMC4767627

[15] B. Yi , Q. Xu , W. Liu , Bioact Mater 2022, 15, 82. 35386347 10.1016/j.bioactmat.2021.12.005 PMC8940767

[16] C. Colosi , S. R. Shin , V. Manoharan , S. Massa , M. Costantini , A. Barbetta , M. R. Dokmeci , M. Dentini , A. Khademhosseini , Adv. Mater. 2016, 28, 677. 26606883 10.1002/adma.201503310 PMC4804470

[17] F. Maiullari , M. Costantini , M. Milan , V. Pace , M. Chirivì , S. Maiullari , A. Rainer , D. Baci , H. E.‐S. Marei , D. Seliktar , C. Gargioli , C. Bearzi , R. Rizzi , Sci. Rep. 2018, 8, 13532. 30201959 10.1038/s41598-018-31848-x PMC6131510

[18] Y. S. Zhang , A. Arneri , S. Bersini , S.‐R. Shin , K. Zhu , Z. Goli‐Malekabadi , J. Aleman , C. Colosi , F. Busignani , V. Dell'Erba , C. Bishop , T. Shupe , D. Demarchi , M. Moretti , M. Rasponi , M. R. Dokmeci , A. Atala , A. Khademhosseini , Biomaterials 2016, 110, 45. 27710832 10.1016/j.biomaterials.2016.09.003 PMC5198581

[19] K. Zhu , S. R. Shin , T. van Kempen , Y.‐C. Li , V. Ponraj , A. Nasajpour , S. Mandla , N. Hu , X. Liu , J. Leijten , Y.‐D. Lin , M. A. Hussain , Y. S. Zhang , A. Tamayol , A. Khademhosseini , Adv. Funct. Mater. 2017, 27, 1605352. 30319321 10.1002/adfm.201605352 PMC6181228

[20] M. Costantini , S. Testa , P. Mozetic , A. Barbetta , C. Fuoco , E. Fornetti , F. Tamiro , S. Bernardini , J. Jaroszewicz , W. Święszkowski , M. Trombetta , L. Castagnoli , D. Seliktar , P. Garstecki , G. Cesareni , S. Cannata , A. Rainer , C. Gargioli , Biomaterials 2017, 131, 98. 28388499 10.1016/j.biomaterials.2017.03.026

[21] M. Costantini , J. Idaszek , K. Szöke , J. Jaroszewicz , M. Dentini , A. Barbetta , J. E. Brinchmann , W. Święszkowski , Biofabrication 2016, 8, 035002. 27431574 10.1088/1758-5090/8/3/035002

[22] M. Özgen Öztürk‐Öncel , B. Hiram Leal‐Martínez , R. F. Monteiro , M. E. Gomes , R. M. A. Domingues , Biomater. Sci. 2023, 11, 5462. 37489648 10.1039/d3bm00626c PMC10408712

[23] L. G. Brunel , S. M. Hull , S. C. Heilshorn , Biofabrication 2022, 14, 032001. 10.1088/1758-5090/ac6bbe PMC10788121 35487196

[24] D. J. Shiwarski , A. R. Hudson , J. W. Tashman , A. W. Feinberg , APL Bioeng. 2021, 5, 010904. 33644626 10.1063/5.0032777 PMC7889293

[25] T. J. Hinton , Q. Jallerat , R. N. Palchesko , J. H. Park , M. S. Grodzicki , H.‐J. Shue , M. H. Ramadan , A. R. Hudson , A. W. Feinberg , Sci. Adv. 2015, 1, e1500758. 26601312 10.1126/sciadv.1500758 PMC4646826

[26] A. Lee , A. R. Hudson , D. J. Shiwarski , J. W. Tashman , T. J. Hinton , S. Yerneni , J. M. Bliley , P. G. Campbell , A. W. Feinberg , Science 2019, 365, 482. 31371612 10.1126/science.aav9051

[27] S. M. Hull , L. G. Brunel , S. C. Heilshorn , Adv. Mater. 2022, 34, 2103691. 10.1002/adma.202103691 PMC8988886 34672027

[28] B. D. Olsen , J. A. Kornfield , D. A. Tirrell , Macromolecules 2010, 43, 9094. 21221427 10.1021/ma101434a PMC3017468

[29] C. D. Lindsay , J. G. Roth , B. L. LeSavage , S. C. Heilshorn , Acta Biomater. 2019, 95, 225. 31096043 10.1016/j.actbio.2019.05.014 PMC7050497

[30] K. Dubbin , Y. Hori , K. K. Lewis , S. C. Heilshorn , Adv. Healthcare Mater. 2016, 5, 2488. 10.1002/adhm.201600636 27581767

[31] L. Raddatz , A. Lavrentieva , I. Pepelanova , J. Bahnemann , D. Geier , T. Becker , T. Scheper , S. Beutel , J. Funct. Biomater. 2018, 9, 63. 30423908 10.3390/jfb9040063 PMC6306849

[32] M. Yeo , J.‐S. Lee , W. Chun , G. H. Kim , Biomacromolecules 2016, 17, 1365. 26998966 10.1021/acs.biomac.5b01764

[33] E. Davoodi , E. Sarikhani , H. Montazerian , S. Ahadian , M. Costantini , W. Swieszkowski , S. M. Willerth , K. Walus , M. Mofidfar , E. Toyserkani , A. Khademhosseini , N. Ashammakhi , Adv. Mater. Technol. 2020, 5, 1901044. 33072855 10.1002/admt.201901044 PMC7567134

[34] I. M. Lei , D. Zhang , W. Gu , J. Liu , Y. Zi , Y. Y. S. Huang , Adv. Mater. Technol. 2023, 8, 2300001.

[35] S. Sakai , K. Mochizuki , Y. Qu , M. Mail , M. Nakahata , M. Taya , Biofabrication 2018, 10, 045007. 30137024 10.1088/1758-5090/aadc9e

[36] A. Basu , A. Saha , C. Goodman , R. T. Shafranek , A. Nelson , ACS Appl. Mater. Interfaces 2017, 9, 40898. 29091399 10.1021/acsami.7b14177

[37] S. Zhang , M. A. Greenfield , A. Mata , L. C. Palmer , R. Bitton , J. R. Mantei , C. Aparicio , M. O. de la Cruz , S. I. Stupp , Nat. Mater. 2010, 9, 594. 20543836 10.1038/nmat2778 PMC3084632

[38] A. Chalard , M. Mauduit , S. Souleille , P. Joseph , L. Malaquin , J. Fitremann , Addit. Manuf. 2020, 33, 101162.

[39] N. Raja , H. Yun , J. Mater. Chem. B 2016, 4, 4707. 32263243 10.1039/c6tb00849f

[40] N. Ashammakhi , A. Hasan , O. Kaarela , B. Byambaa , A. Sheikhi , A. K. Gaharwar , A. Khademhosseini , Adv. Healthcare Mater. 2019, 8, e1801048. 10.1002/adhm.201801048 30734530

[41] Y. Jin , A. Compaan , T. Bhattacharjee , Y. Huang , Biofabrication 2016, 8, 025016. 27257095 10.1088/1758-5090/8/2/025016

[42] Y. He , F. Yang , H. Zhao , Q. Gao , B. Xia , J. Fu , Sci. Rep. 2016, 6, 29977. 27436509 10.1038/srep29977 PMC4951698

[43] J. Wang , Y. Liu , X. Zhang , S. E. Rahman , S. Su , J. Wei , F. Ning , Z. Hu , R. Martínez‐Zaguilán , S. R. Sennoune , W. Cong , G. Christopher , K. Zhang , J. Qiu , Polymer 2021, 214, 123238.

[44] J. P. K. Armstrong , M. Burke , B. M. Carter , S. A. Davis , A. W. Perriman , Adv. Healthcare Mater. 2016, 5, 1724. 10.1002/adhm.201600022 27125336

[45] A. L. Rutz , K. E. Hyland , A. E. Jakus , W. R. Burghardt , R. N. Shah , Adv. Mater. 2015, 27, 1607. 25641220 10.1002/adma.201405076 PMC4476973

[46] T. Hu , X. Cui , M. Zhu , M. Wu , Y. Tian , B. Yao , W. Song , Z. Niu , S. Huang , X. Fu , Bioact Mater 2020, 5, 808. 32637745 10.1016/j.bioactmat.2020.06.001 PMC7317699

[47] Y. Zhu , C. J. Stark , S. Madira , S. Ethiraj , A. Venkatesh , S. Anilkumar , J. Jung , S. Lee , C. A. Wu , S. K. Walsh , G. A. Stankovich , Y.‐P. J. Woo , Bioengineering 2022, 9, 807. 36551013 10.3390/bioengineering9120807 PMC9774270

[48] J. Leppiniemi , P. Lahtinen , A. Paajanen , R. Mahlberg , S. Metsä‐Kortelainen , T. Pinomaa , H. Pajari , I. Vikholm‐Lundin , P. Pursula , V. P. Hytönen , ACS Appl. Mater. Interfaces 2017, 9, 21959. 28598154 10.1021/acsami.7b02756

[49] J. Hammer , L.‐H. Han , X. Tong , F. Yang , Tissue Eng., Part C 2014, 20, 169. 10.1089/ten.TEC.2013.0176 23745610

[50] H. Wen , J. Li , G. F. Payne , Q. Feng , M. Liang , J. Chen , H. Dong , X. Cao , Biofabrication 2020, 12, 035007. 32155609 10.1088/1758-5090/ab7e74

[51] A. Sydney Gladman , E. A. Matsumoto , R. G. Nuzzo , L. Mahadevan , J. A. Lewis , Nat. Mater. 2016, 15, 413. 26808461 10.1038/nmat4544

[52] J. Lai , X. Ye , J. Liu , C. Wang , J. Li , X. Wang , M. Ma , M. Wang , Mater. Des. 2021, 205, 109699.

[53] A. Kirillova , R. Maxson , G. Stoychev , C. T. Gomillion , L. Ionov , Adv. Mater. 2017, 29, 1703443. 10.1002/adma.201703443 29024044

[54] J. Gong , C. Schuurmans , A. M. van Genderen , X. Cao , W. Li , F. Cheng , J. He , A. López , V. Huerta , J. Manriquez , R. Li , H. Li , C. Delavaux , S. Sebastian , P. Capendale , H. Wang , J. Xie , M. Yu , R. Masereeuw , Y. S. Zhang , Nat. Commun. 2020, 11, 1267. 32152307 10.1038/s41467-020-14997-4 PMC7062888

[55] C. Aronsson , M. Jury , S. Naeimipour , F. R. Boroojeni , J. Christoffersson , P. Lifwergren , C.‐F. Mandenius , R. Selegård , D. Aili , Biofabrication 2020, 12, 035031. 32428894 10.1088/1758-5090/ab9490

[56] J. Malda , J. Visser , F. P. Melchels , T. Jüngst , W. E. Hennink , W. J. A. Dhert , J. Groll , D. W. Hutmacher , Adv. Mater. 2013, 25, 5011. 24038336 10.1002/adma.201302042

[57] K. Schütz , A.‐M. Placht , B. Paul , S. Brüggemeier , M. Gelinsky , A. Lode , J. Tissue Eng. Regener. Med. 2017, 11, 1574. 10.1002/term.2058 26202781

[58] H. Li , Y. J. Tan , K. F. Leong , L. Li , ACS Appl. Mater. Interfaces 2017, 9, 20086. 28530091 10.1021/acsami.7b04216

[59] T. Ahlfeld , N. Cubo‐Mateo , S. Cometta , V. Guduric , C. Vater , A. Bernhardt , A. R. Akkineni , A. Lode , M. Gelinsky , ACS Appl. Mater. Interfaces 2020, 12, 12557. 32092249 10.1021/acsami.0c00710

[60] S. Liu , D. Kilian , T. Ahlfeld , Q. Hu , M. Gelinsky , Biofabrication 2023, 15, 025013. 10.1088/1758-5090/acb8dc 36735961

[61] C. Czichy , D. Kilian , T.‐C. Wang , S. Günther , A. Lode , M. Gelinsky , S. Odenbach , J. Mech. Behav. Biomed. Mater. 2022, 131, 105253. 35490511 10.1016/j.jmbbm.2022.105253

[62] S. M. Hull , J. Lou , C. D. Lindsay , R. S. Navarro , B. Cai , L. G. Brunel , A. D. Westerfield , Y. Xia , S. C. Heilshorn , Sci. Adv. 2023, 9, eade7880. 37000873 10.1126/sciadv.ade7880 PMC10065439

[63] C. D. Devillard , C. A. Mandon , S. A. Lambert , L. J. Blum , C. A. Marquette , Biotechnol. J. 2018, 13, 1800098. 10.1002/biot.201800098 30192055

[64] K. W. Rogers , A. F. Schier , Annu. Rev. Cell Dev. Biol. 2011, 27, 377. 21801015 10.1146/annurev-cellbio-092910-154148

[65] B. N. Johnson , K. Z. Lancaster , G. Zhen , J. He , M. K. Gupta , Y. L. Kong , E. A. Engel , K. D. Krick , A. Ju , F. Meng , L. W. Enquist , X. Jia , M. C. McAlpine , Adv. Funct. Mater. 2015, 25, 6205. 26924958 10.1002/adfm.201501760 PMC4765385

[66] D. Kilian , S. Cometta , A. Bernhardt , R. Taymour , J. Golde , T. Ahlfeld , J. Emmermacher , M. Gelinsky , A. Lode , Biofabrication 2022, 14, 014108. 10.1088/1758-5090/ac457b 34933296

[67] A. Di Luca , M. Klein‐Gunnewiek , J. G. Vancso , C. A. van Blitterswijk , E. M. Benetti , L. Moroni , Biotechnol. J. 2017, 12, 1700072. 10.1002/biot.201700072 28865136

[68] B. T. Smith , S. M. Bittner , E. Watson , M. M. Smoak , L. Diaz‐Gomez , E. R. Molina , Y. S. Kim , C. D. Hudgins , A. J. Melchiorri , D. W. Scott , K. J. Grande‐Allen , J. J. Yoo , A. Atala , J. P. Fisher , A. G. Mikos , Tissue Eng., Part A 2020, 26, 239. 31696784 10.1089/ten.tea.2019.0204 PMC7133451

[69] A. W. James , G. LaChaud , J. Shen , G. Asatrian , V. Nguyen , X. Zhang , K. Ting , C. Soo , Tissue Eng., Part B Rev. 2016, 22, 284. 26857241 10.1089/ten.teb.2015.0357 PMC4964756

[70] F. E. Freeman , P. Pitacco , L. H. A. van Dommelen , J. Nulty , D. C. Browe , J.‐Y. Shin , E. Alsberg , D. J. Kelly , Sci. Adv. 2020, 6, eabb5093. 32851179 10.1126/sciadv.abb5093 PMC7428335

[71] Y. Sun , Y. You , W. Jiang , B. Wang , Q. Wu , K. Dai , Sci. Adv. 2020, 6, eaay1422. 32917692 10.1126/sciadv.aay1422 PMC11206535

[72] Y. Ou , S. Cao , Y. Zhang , H. Zhu , C. Guo , W. Yan , F. Xin , W. Dong , Y. Zhang , M. Narita , Z. Yu , T. P. J. Knowles , Nat. Commun. 2023, 14, 322. 36658120 10.1038/s41467-022-35140-5 PMC9852579

[73] D. B. Kolesky , K. A. Homan , M. A. Skylar‐Scott , J. A. Lewis , Proc. Natl. Acad. Sci. USA 2016, 113, 3179. 26951646 10.1073/pnas.1521342113 PMC4812707

[74] Q. Li , Z. Jiang , L. Ma , J. Yin , Z. Gao , L. Shen , H. Yang , Z. Cui , H. Ye , H. Zhou , Biofabrication 2022, 14, 035022. 10.1088/1758-5090/ac7909 35705061

[75] W. Liu , Z. Zhong , N. Hu , Y. Zhou , L. Maggio , A. K. Miri , A. Fragasso , X. Jin , A. Khademhosseini , Y. S. Zhang , Biofabrication 2018, 10, 024102. 29176035 10.1088/1758-5090/aa9d44 PMC5837947

[76] S. M. Hull , C. D. Lindsay , L. G. Brunel , D. J. Shiwarski , J. W. Tashman , J. G. Roth , D. Myung , A. W. Feinberg , S. C. Heilshorn , Adv. Funct. Mater. 2021, 31, 2007983. 33613150 10.1002/adfm.202007983 PMC7888563

[77] A. Urciuolo , G. G. Giobbe , Y. Dong , F. Michielin , L. Brandolino , M. Magnussen , O. Gagliano , G. Selmin , V. Scattolini , P. Raffa , P. Caccin , S. Shibuya , D. Scaglioni , X. Wang , J. Qu , M. Nikolic , M. Montagner , G. L. Galea , H. Clevers , M. Giomo , P. De Coppi , N. Elvassore , Nat. Commun. 2023, 14, 3128. 37253730 10.1038/s41467-023-37953-4 PMC10229611

[78] Q. Gao , Y. He , J. Fu , A. Liu , L. Ma , Biomaterials 2015, 61, 203. 26004235 10.1016/j.biomaterials.2015.05.031

[79] Y. Zhang , Y. Yu , H. Chen , I. T. Ozbolat , Biofabrication 2013, 5, 025004. 23458889 10.1088/1758-5082/5/2/025004 PMC4281173

[80] F. Dolati , Y. Yu , Y. Zhang , A. M. D. Jesus , E. A. Sander , I. T. Ozbolat , Nanotechnology 2014, 25, 145101. 24632802 10.1088/0957-4484/25/14/145101 PMC4281171

[81] H. Cui , W. Zhu , Y. Huang , C. Liu , Z.‐X. Yu , M. Nowicki , S. Miao , Y. Cheng , X. Zhou , S.‐J. Lee , Y. Zhou , S. Wang , M. Mohiuddin , K. Horvath , L. G. Zhang , Biofabrication 2019, 12, 015004. 31470437 10.1088/1758-5090/ab402c PMC6803062

[82] L. Ouyang , J. A. Burdick , W. Sun , ACS Appl. Mater. Interfaces 2018, 10, 12424. 29582989 10.1021/acsami.7b19537

[83] Y. Zhou , Q. Gui , W. Yu , S. Liao , Y. He , X. Tao , Y. Yu , Y. Wang , ACS Biomater. Sci. Eng. 2019, 5, 6311. 33405538 10.1021/acsbiomaterials.9b01293

[84] A. G. Savelyev , A. V. Sochilina , R. A. Akasov , A. V. Mironov , A. Y. Kapitannikova , T. N. Borodina , N. V. Sholina , K. V. Khaydukov , A. V. Zvyagin , A. N. Generalova , E. V. Khaydukov , Front. Bioeng. Biotechnol. 2021, 9, 783834. 34926429 10.3389/fbioe.2021.783834 PMC8678487

[85] Y. Wu , G. M. Fortunato , B. O. Okesola , F. L. P. D. Brocchetti , R. Suntornnond , J. Connelly , C. D. Maria , J. C. Rodriguez‐Cabello , G. Vozzi , W. Wang , A. Mata , Biofabrication 2021, 13, 035027. 10.1088/1758-5090/abe4c3 33561850

[86] S. Zhang , C. Qi , W. Zhang , H. Zhou , N. Wu , M. Yang , S. Meng , Z. Liu , T. Kong , Adv. Mater. 2023, 35, 2209263. 10.1002/adma.202209263 36448877

[87] S. Shin , L. G. Brunel , B. Cai , D. Kilian , J. G. Roth , A. J. Seymour , S. C. Heilshorn , Adv. Funct. Mater. 2023, 33, 2307435. 10.1002/adfm.202307435 PMC11031202 38646474

[88] H. J. V. Tyrrell , K. R. Harris , Diffusion in Liquids, Elsevier, New York 1984.

[89] C. C. Miller , Proc. Royal Soc. London A 1924, 106, 724.

[90] E. Axpe , D. Chan , G. S. Offeddu , Y. Chang , D. Merida , H. L. Hernandez , E. A. Appel , Macromolecules 2019, 52, 6889. 31579160 10.1021/acs.macromol.9b00753 PMC6764024

[91] S. R. Lustig , N. A. Peppas , J. Appl. Polym. Sci. 1988, 36, 735.

[92] N. R. Richbourg , A. Ravikumar , N. A. Peppas , Macromol. Chem. Phys. 2021, 222, 2100138. 34456531 10.1002/macp.202100138 PMC8389770

[93] L. Riley , P. Cheng , T. Segura , Nat. Comput. Sci. 2023, 3, 975. 38177603 10.1038/s43588-023-00551-x

[94] C. M. Franca , A. Athirasala , R. Subbiah , A. Tahayeri , P. Selvakumar , A. Mansoorifar , S. Horsophonphong , A. Sercia , L. Nih , L. E. Bertassoni , Adv. Healthcare Mater. 2023, 12, 2202840. 10.1002/adhm.202202840 PMC10526736 37219011

[95] A. R. Anderson , E. Nicklow , T. Segura , Acta Biomater. 2022, 150, 111. 35917913 10.1016/j.actbio.2022.07.051 PMC10329855

[96] J. T. Peters , M. E. Wechsler , N. A. Peppas , Regen. Biomater. 2021, 8, rbab060. 34925879 10.1093/rb/rbab060 PMC8678442

[97] K. Vulic , M. S. Shoichet , Biomacromolecules 2014, 15, 3867. 25230248 10.1021/bm501084u

[98] S. Correa , A. K. Grosskopf , H. Lopez Hernandez , D. Chan , A. C. Yu , L. M. Stapleton , E. A. Appel , Chem. Rev. 2021, 121, 11385. 33938724 10.1021/acs.chemrev.0c01177 PMC8461619

[99] B. Marco‐Dufort , M. W. Tibbitt , Mater. Today Chem. 2019, 12, 16.

[100] J. Su , F. Chen , V. L. Cryns , P. B. Messersmith , J. Am. Chem. Soc. 2011, 133, 11850. 21751810 10.1021/ja203077x PMC3149454

[101] H. Ding , R. C. Chang , Appl. Sci. 2018, 8, 403.

[102] W. Wu , A. DeConinck , J. A. Lewis , Adv. Mater. 2011, 23, H178. 21438034 10.1002/adma.201004625

[103] G. Palazzo , H. Mateos , D. Berti , in Colloidal Foundations of Nanoscience, 2nd Edition, (Eds: D. Berti , G. Palazzo ), Elsevier, Amsterdam, 2022, pp. 201–225.

[104] S. Sheth , E. Barnard , B. Hyatt , M. Rathinam , S. P. Zustiak , Front. Bioeng. Biotechnol. 2019, 7, 410. 31956651 10.3389/fbioe.2019.00410 PMC6951421

[105] B. Amsden , Macromolecules 1998, 31, 8382.

[106] M. Ryma , H. Genç , A. Nadernezhad , I. Paulus , D. Schneidereit , O. Friedrich , K. Andelovic , S. Lyer , C. Alexiou , I. Cicha , J. Groll , Adv. Mater. 2022, 34, 2200653. 10.1002/adma.202200653 35595711

[107] G. S. Offeddu , L. Mohee , R. E. Cameron , J. Mater. Sci.: Mater. Med. 2020, 31, 46. 32367247 10.1007/s10856-020-06381-x PMC7198636

[108] V. Hagel , T. Haraszti , H. Boehm , Biointerphases 2013, 8, 36. 24706145 10.1186/1559-4106-8-36

[109] A. Rahmatpour , N. Alijani , A. Mirkani , React. Funct. Polym. 2023, 185, 105537.

[110] W. Wang , S. A. Sande , Polym. J. 2015, 47, 302.

[111] R. L. Blackmon , S. M. Kreda , P. R. Sears , L. E. Ostrowski , D. B. Hill , B. S. Chapman , J. B. Tracy , A. L. Oldenburg , Proc. SPIE Int. Soc. Opt. Eng. 2016, 9697, 969724. 27746581 10.1117/12.2208805 PMC5061133

[112] J. Lee , W. Wu , J. Y. Jiang , B. Zhu , D. A. Boas , Opt. Express 2012, 20, 22262. 23037374 10.1364/OE.20.022262 PMC3601731

[113] P. I. Lee , J. Controlled Release 1985, 2, 277.

[114] M. Vigata , C. Meinert , D. W. Hutmacher , N. Bock , Pharmaceutics 2020, 12, 1188. 33297493 10.3390/pharmaceutics12121188 PMC7762425

[115] M. Machour , N. Hen , I. Goldfracht , D. Safina , M. Davidovich‐Pinhas , H. Bianco‐Peled , S. Levenberg , Adv. Sci. 2022, 9, 2200882. 10.1002/advs.202200882 PMC9731703 36261395

[116] J. G. Roth , L. G. Brunel , M. S. Huang , Y. Liu , B. Cai , S. Sinha , F. Yang , S. P. Pașca , S. Shin , S. C. Heilshorn , Nat. Commun. 2023, 14, 4346. 37468483 10.1038/s41467-023-40006-5 PMC10356773

[117] M. H. Hettiaratchi , A. Schudel , T. Rouse , A. J. García , S. N. Thomas , R. E. Guldberg , T. C. McDevitt , APL Bioeng. 2018, 2, 026110. 31069307 10.1063/1.4999925 PMC6324205

[118] B. Mirani , E. Stefanek , B. Godau , S. M. Hossein Dabiri , M. Akbari , ACS Biomater. Sci. Eng. 2021, 7, 3269. 34142796 10.1021/acsbiomaterials.1c00084

[119] U. Akalp , S. J. Bryant , F. J. Vernerey , Soft Matter 2016, 12, 7505. 27548744 10.1039/c6sm00583g PMC5341105

[120] H. A. Leddy , H. A. Awad , F. Guilak , J. Biomed. Mater. Res. 2004, 70B, 397. 10.1002/jbm.b.30053 15264325

[121] A. Bläßle , G. Soh , T. Braun , D. Mörsdorf , H. Preiß , B. M. Jordan , P. Müller , Nat. Commun. 2018, 9, 1582. 29679054 10.1038/s41467-018-03975-6 PMC5910415

[122] M. L. Macdonald , R. E. Samuel , N. J. Shah , R. F. Padera , Y. M. Beben , P. T. Hammond , Biomaterials 2011, 32, 1446. 21084117 10.1016/j.biomaterials.2010.10.052 PMC3033887

[123] X. Li , B. Liu , B. Pei , J. Chen , D. Zhou , J. Peng , X. Zhang , W. Jia , T. Xu , Chem. Rev. 2020, 120, 10793. 32902959 10.1021/acs.chemrev.0c00008

[124] S. M. Montgomery , C. M. Hamel , J. Skovran , H. J. Qi , Extreme Mech. Lett. 2022, 53, 101714.

[125] D. Ribezzi , M. Gueye , S. Florczak , F. Dusi , D. de Vos , F. Manente , A. Hierholzer , M. Fussenegger , M. Caiazzo , T. Blunk , J. Malda , R. Levato , Adv. Mater. 2023, 35, 2301673. 10.1002/adma.202301673 37269532

[126] O. Lieleg , K. Ribbeck , Trends Cell Biol. 2011, 21, 543. 21727007 10.1016/j.tcb.2011.06.002 PMC3164742

[127] M. Kuzucu , G. Vera , M. Beaumont , S. Fischer , P. Wei , V. P. Shastri , A. Forget , ACS Biomater. Sci. Eng. 2021, 7, 2192. 33970597 10.1021/acsbiomaterials.1c00183 PMC8207502

[128] J. Xu , Y.‐L. Tsai , S. Hsu , Molecules 2020, 25, 5296. 33202861 10.3390/molecules25225296 PMC7698101

[129] D. Kilian , W. Kilian , A. Troia , T.‐D. Nguyen , B. Ittermann , L. Zilberti , M. Gelinsky , ACS Appl. Mater. Interfaces 2022, 14, 48397. 36270624 10.1021/acsami.2c12872 PMC9634698

[130] E. Natsaridis , P. Mouzoura , F. Gkartziou , A. Marazioti , S. G. Antimisiaris , Int. J. Dev. Biol. 2022, 66, 137. 34549789 10.1387/ijdb.210108sa

[131] C. F. Ceballos‐González , E. J. Bolívar‐Monsalve , D. A. Quevedo‐Moreno , L. L. Lam‐Aguilar , K. I. Borrayo‐Montaño , J. F. Yee‐de León , Y. S. Zhang , M. M. Alvarez , G. Trujillo‐de Santiago , ACS Biomater. Sci. Eng. 2021, 7, 2408. 33979127 10.1021/acsbiomaterials.0c01646

[132] A. Hajikhani , F. Scocozza , M. Conti , M. Marino , F. Auricchio , P. Wriggers , Int. J. Artif. Organs 2019, 42, 548. 31267806 10.1177/0391398819856024

[133] L. Zhong , J.‐M. Zhang , B. Su , R. S. Tan , J. C. Allen , G. S. Kassab , Front. Physiol. 2018, 9, 742. 29997520 10.3389/fphys.2018.00742 PMC6028770

[134] N.‐U. Kang , S.‐J. Lee , S.‐J. Gwak , Yonsei Med. J. 2022, 63, 114. 35083896 10.3349/ymj.2022.63.2.114 PMC8819402

